# Chromosome-level assembly of *Lemnamoeba sinaloensis* gen. nov. et sp. nov. (Apofilosa) reveals dynamic genome evolution in Rhizaria

**DOI:** 10.1186/s12864-026-13502-3

**Published:** 2026-10-28

**Authors:** Markus Hiltunen Thorén, Ioana Onuț-Brännström, Vasily Zlatogursky, Yash Pardasani, Hana Pecková, Anežka Karlíková, Astrid S. Holzer, Fabien Burki

**Affiliations:** 1 Department of Organismal Biology, Systematic biology, Uppsala University, Norbyv. 18 D, Uppsala, 752 36 Sweden; 2 Department of Ecology, Environment and Plant Sciences, Stockholm University, Svante Arrhenius v. 20 A, 114 18 Stockholm, Sweden; 3 The Royal Swedish Academy of Sciences, Lilla Frescativ. 4 A, 114 18 Stockholm, Sweden; 4 Department of Ecology and Genetics, Program in Evolutionary Biology, Uppsala University, Norbyv. 18 D, Uppsala, 752 36 Sweden; 5 Institute of Parasitology, Biology Centre of the Czech Academy of Sciences, Branišovská 1160/31, České Budějovice, 370 05 Czech Republic; 6 Department of Parasitology, Faculty of Sciences, University of South Bohemia, Branišovská 1645/31a, České Budějovice, 370 05 Czech Republic; 7 Fish Health Division, Clinical Department of Farm Animals and Food System Transformation, University of Veterinary Medicine, Veterinärplatz 1, Vienna, 1210 Austria

## Abstract

**Background:**

Rhizaria is a eukaryotic supergroup including many amoeboid protists. Despite harboring a large diversity and many ecologically important species, the genomes belonging to this supergroup remain poorly explored. With only a handful available genome sequences, most of low contiguity, little is known about overall architecture, characteristics, and evolution of rhizarian genomes.

**Results:**

We provide a highly contiguous genome assembly of an undescribed marine rhizarian amoeba formerly known as M6MM. The assembly revealed a small (27 Mb; 8704 genes) and compact genome (84% genes, 10% repeats, 6% intergenic regions), and was almost fully captured in six contigs confirmed cytologically to represent chromosomes. The genome contains a very large amount of simple-sequence repeats (SSRs), particularly of the AC:GT dinucleotide class (206 loci/Mb), in total covering almost 2% of the assembly. We also obtained the complete mitochondrial genome, which is reduced and devoid of ribosomal protein genes similarly to some other rhizarians. Some of these “missing” mitochondrial-encoded genes are found in the nuclear genome and are predicted to be targeted to the mitochondrion. In our 18S rDNA phylogenetic analysis, M6MM loosely groups with environmental sequences and the amoeboflagellate *Viscidocauda repens*. However, this amoeba is phylogenetically distant from any described species and expresses a unique pseudopodial morphology, prompting us to erect the novel genus and species *Lemnamoeba sinaloensis* gen. nov. et. sp. nov. Environmental data show that *L. sinaloensis* has a wide marine distribution and can mostly be found between the surface and 100 m depths.

**Conclusion:**

This study adds another genus to the hitherto taxonomically poor Apofilosa, provides one of the highest-quality rhizarian genome assemblies to date, and reveals an abundance of SSRs in the genome, highlighting the complexity of eukaryotic genomes in underexplored parts of the tree of life.

**Supplementary Information:**

The online version contains supplementary material available at https://doi.org/10.1186/s12864-026-13502-3.

## Introduction

Rhizaria is a major group of mostly single-celled eukaryotes that are found in almost all environments. It comprises a vast diversity of forms and functions, from charismatic amoebae such as radiolarians, to testate reticulopodiate foraminiferans, many filose cells and countless flagellates and amoeboflagellates [1, 2]. While almost all rhizarians are bacteri- or eukaryvorous predators [3–5], including specialized behavior such as protoplast feeding, the group also comprises photosynthetic lineages (chlorarachniophytes and *Paulinella*) that have been important models to study plastid evolution [6, 7]. Rhizaria also contain parasitic groups, including economically important pests such as the agent responsible for the clubroot disease or several pathogens of shellfish [8–10]. Although most rhizarians are microscopic, examples of giant single-cell organisms are known, such as the testate filose amoeba *Gromia* that can reach up to a few centimeters [11]. Collectively, rhizarian cells make up for about half of the total biomass of all other mesozooplankton in the oligotrophic intertropical open oceans [12], and play important roles in soil [13].

The phylogeny of Rhizaria has been investigated in some detail, including in recent years using multigene phylogenomic datasets [5, 9, 14, 15]. While many open questions remain, for example the validation of some of the main proposed groups such as Cercozoa, the latest phylogenomic trees have consistently recovered a few large assemblages. These are Retaria, which groups radiolarians and foraminiferans together, the mainly filose and flagellated cells in Filosa, and the animal and plant/algal parasites in Ascetosporea and Phytomyxea, respectively [5, 8, 9, 15, 16]. In addition, Apofilosa is a smaller group of amoeboid protists that currently only includes the two genera *Gromia* and *Filoreta*, as well as the undescribed M6MM (see below) [9].

Despite the importance of Rhizaria as a major eukaryotic group, it remains poorly investigated from a genomic perspective [1, 15, 17]. Draft genomes are available from the filosans *Bigelowiella natans*,* Paulinella micropora*, and *Amorphochlora amoebiformis*, the foraminifer *Reticulomyxa filosa*, the phytomyxean parasites *Plasmodiophora brassicae* and *Spongospora subterranea*, and a few ascetosporean parasites [6, 9, 10, 18–22]. Not only are there few rhizarian genomes available, but most were assembled from short Illumina reads, which resulted in a low genome contiguity (hundreds to thousands of scaffolds and many sequencing gaps) and miss information on the genome architecture. To date, only the genome assembly of the parasite *Pl. brassicae* is of chromosome-level quality, and this has led to important findings about the chromosome numbers, repetitive content and life cycle [19]. To be able to establish a complete picture of the structure and function of a genome, and the genomic evolution of a major eukaryotic group such as Rhizaria, a diversity of high-quality genomes is needed [23, 24].

**Fig. 1:**
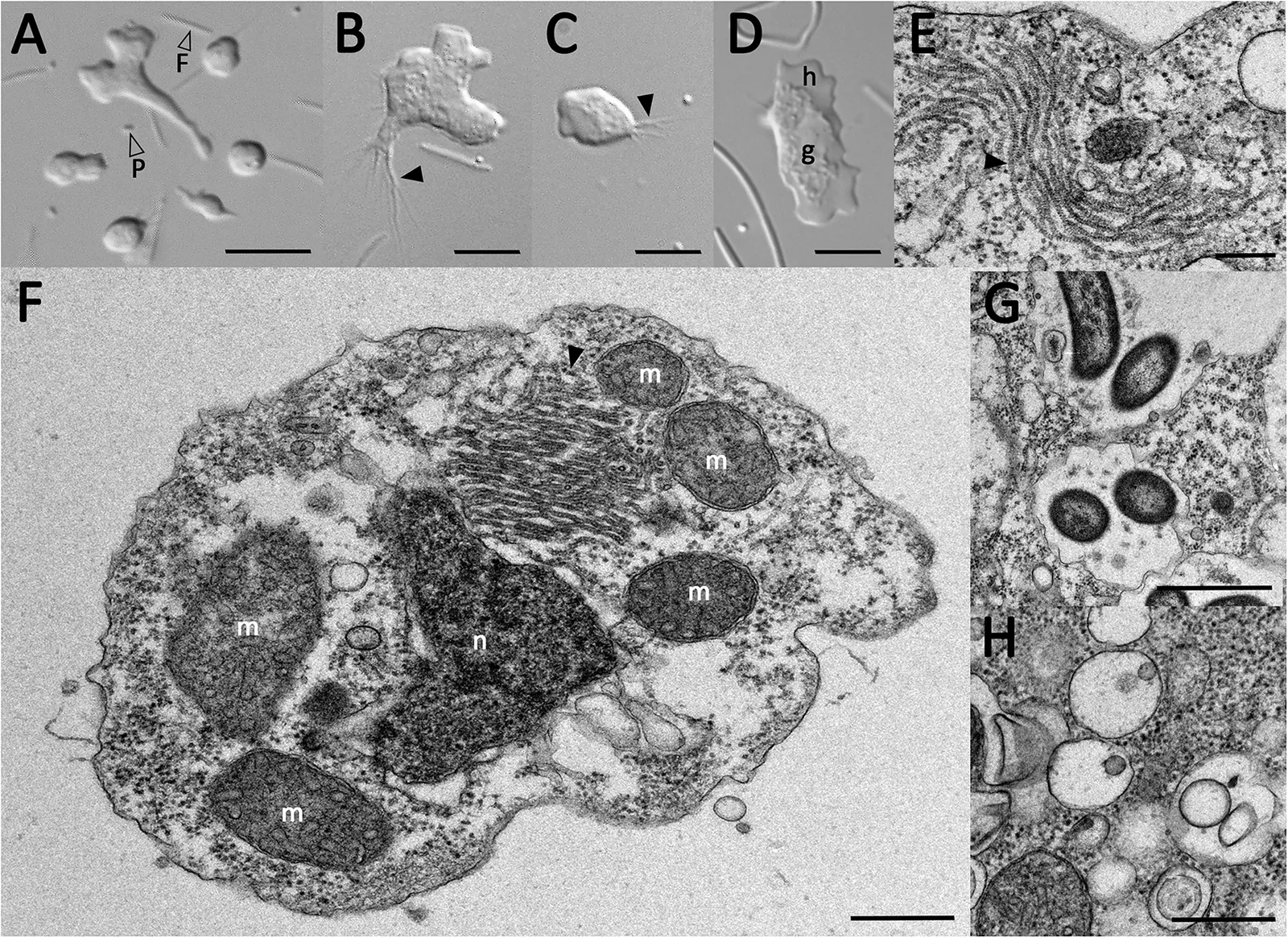
Light and electron microscopic organisation of *Lemnamoeba sinaloensis* cells. **A-D**. Light microscopic images of amoebae in hanging drop preparation and under DIC. **A**. co-culture of variably shaped amoebae and two co-isolated bacteria, identified as *Flammeovirga* sp. (F; long rods) and *Pseudorhodobacter* sp. (P; short rods) by genome sequencing. **B-C**. Amoebae of different sizes with typical adhesive uroidal structures (black arrowheads). **D**. Amoeba showing clear differentiation between smooth hyaloplasm (h) and granuloplasm (g). **E-H**. TEM of *L. sinaloensis*. **E**. Characteristic ribbon-like structure of packed tubules (black arrowhead) in the cytoplasm. **F**. Cross-section through amoeba showing mitochondria with tubular cristae (m), nucleus (n), and packed tubules (black arrowhead). **G**. Food vacuoles containing rod-shaped bacteria (dark structures, cross and transverse sections). **H**. Late-stage food vacuoles containing membrane-within-membrane remnants. Size bars: A. 15 μm, B-D. 5 μm, E. 200 nm, F. 500 nm, G-H. 400 nm. A video recording of cells in movement can be found in Additional File 1

Recently, we sequenced and assembled a draft genome of the strain M6MM, an amoeboid protist isolated from the gills of a tropical marine fish [9]. Using a phylogenomic approach, the amoeba was inferred to belong to Rhizaria and clustered with Apofilosa [9]. However, this draft genome was assembled from short-read data and contained a high number of scaffolds, which appeared to be caused by an unusual amount of simple-sequence repeats (SSRs) that occurred at the beginning and end of each contig. Here, we formally describe M6MM as *Lemnamoeba sinaloensis* gen. nov. et sp. nov. (Fig. 1), and use long-read genome sequencing to generate a near-complete version of its genome. Facilitated by this improved assembly, we provide an overview of cytoskeletal and motor proteins encoded in its genome, a detailed analysis of the SSRs, and characterize a complete mitogenome. Finally, we complement the existing phylogenomic tree with an 18S rDNA phylogeny, placing *L. sinaloensis* in a wider diversity context, and infer its geographic distribution and abundance using the global EukBank dataset.

## Results

### A high-quality genome assembly of *Lemnamoeba sinaloensis* gen. nov. et sp. nov

PacBio sequencing resulted in a total of 25.8 billion bases in HiFi reads, with an average read length of 9.6 kbp. After an initial assembly was created, bacterial contigs were identified based on divergent GC% (clearly lower or higher than the main contigs) and depth of coverage. Bacterial contigs were classified as *Pseudorhodobacter* sp. (α-Proteobacteria) and *Flammeovirga* sp. (Bacteroidetes) based on percent identity of the 16S ribosomal DNA sequence when compared to GenBank (both > 99% sequence identity). Both bacterial genomes were represented as two circular chromosomes, with the addition of a plasmid in *Flammeovirga* sp. Gram staining and TEM confirmed the presence of two morphologically distinct rod-shaped bacterial strains, one elongated Gram-negative strain (*Flammeovirga* sp.) and one more ovoid Gram-positive strain (*Pseudorhodobacter* sp.) (Fig. 1A). Cells of both strains were commonly observed in the culture medium and within digestive vacuoles of *L. sinaloensis* (Fig. 1G), and thus probably represent a food source for the amoebae, rather than endosymbionts. It is likely that the bacteria were co-isolated from seawater or form part of the microbiome of the finescale triggerfish, from which the amoebae were isolated.

Assembly and curation of the *L. sinaloensis* genome resulted in a 27 Mb assembly consisting of 15 contigs, with the six longest contigs covering > 98% of the assembly and likely corresponding to the chromosomes (N50 = 5,826,847 bp; Table 1). Supporting this, cytological analysis of metaphase cells showed six large chromosomes ranging in size between ca. 2.35–5.59 µm (Fig. S1, Table S1). Genome size estimate based on k-mers from the HiFi reads resulted in 22 Mb, similar to the assembly size. The k-mer based analysis also predicted > 99% of the genome to be homozygous, suggesting the amoeba was in a haploid state when harvested. The GC content of the genome assembly was 53.2%. No canonical telomere repeat sequences (5’-TTAGGG-3’ at the 3’ end) could be detected. Instead, a repetitive motif consisting of TAA[G]_n_ was found in five cases, including the reverse complement at the 5’ end; however, this sequence was also identified within contigs and may not be unique to telomeres.

Among Rhizaria, the genome size of *L. sinaloensis* is relatively small with a low number of genes (Table 1). It is more compact than most other rhizarian genomes, with a short average intergenic distance, while the number of introns per gene is high. The canonical splice sites (GT/AG) were present in 99.5% of introns, but we also found GC/AG in 0.2%, with the remaining 0.3% formed by other base combinations. The full set of tRNA genes was detected in the nuclear genome, corresponding to a total of 461 genes. Functional gene annotation resulted in a total of 6568 annotated genes (80%, not counting tRNAs). While Rhizaria is not available as a separate lineage in OrthoDB, BUSCO analysis against the alveolata_odb12 lineage dataset revealed high completeness (5 missing out of 99 BUSCOs in the proteome; Fig. S2).

**Table 1:** Statistics of sequenced rhizarian genomes. ‘Total repeat content’ includes simple-sequence repeats, interspersed repeats, and other repetitive DNA. ‘Unknown’: Not reported in the reference and gene annotation unavailable

Species	Assembly size (Mb)	Num. Scaffolds	N50	L50	Gene number	Avg. intron number	Avg. intron length (bp)	Median intergenic distance (bp)	Total repeat content (%)	Reference
*Lemnamoeba sinaloensis*	27.0	15	5,826,847	2	8 704	13	82	162	10.1	This paper
*Bigelowiella natans*	94.7	302	819,951	36	21 708	8	182	960	12.9	Curtis et al. (2012) [6]
*Reticulomyxa filosa*	101.9	50,809	3609	7416	39 965	2	87	426	30.5	Glöckner et al. (2014) [18]
*Paulinella micropora*	707.1	7048	143,028	1555	32 361	10	882	Unknown	82.3	Lhee et al. (2021) [20]
*Spongospora subterranea*	31.5	346	122,734	73	10 325	Unknown	Unknown	Unknown	27.4	Arjarquah et al. (2025) [21]
*Plasmodiophora brassicae*	25.2	20	1,360,625	8	9 230	4	68	276	11.3	Stjelja et al. (2019) [19]
*Amorphochlora amoebiformis*	214.2	567	693,001	98	17 610	16	550	670	68.6	Aoki et al. (2025) [22]

### High numbers of actin and kinesin genes

The motility of single-celled amoeboid organisms such as *L. sinaloensis* is governed by the actin filaments and microtubules of the cytoskeleton. Comparative genomics of Rhizaria have revealed interesting patterns of cytoskeleton as well as motor protein evolution, e.g., duplication and neofunctionalization of β-tubulin in Foraminifera [18, 25, 26]. We screened the *L. sinaloensis* genome for homologs of cytoskeleton and motor protein coding genes, and identified most canonical genes (Table 2, Table S2). In particular, eleven actin paralogs were detected, showing high nucleotide similarity to each other, suggesting recent gene duplication events (Fig. S3; 10% variable sites, average number of pairwise differences = 0.038). Thirty kinesin genes were found, including members of eight out of 14 families [27], and 12 genes that could not be assigned to a family and may represent orphan genes (Fig. S4). In other Rhizaria, particularly Foraminifera and Filosa, the polyubiquitin monomer-monomer junctions contain one or two amino acid insertions [28, 29]. We identified this gene in *L. sinaloensis* and found that the junction contained an insertion of asparagine and glycine (NG), similarly as in *Massisteria marina* (Filosa), but unlike the inferred relative *Gromia oviformis* (Apofilosa), which has a single serine (S) insertion [28].

**Table 2:** Cytoskeleton and motor proteins encoded in the *Lemnamoeba sinaloensis* genome. See Table S2 for a full list of gene identifiers, Fig. S3 for the actin and Fig. S4 for the kinesin phylogenies

Gene family	Members	Organization
**Cytoskeleton**		
⍺-tubulin	4	alpha1 type [26].
ꞵ-tubulin	2	Both non-foraminiferan [25].
actin	11	
⍺-actinin	1	Two predicted isoforms.
**Motor proteins**		
myosin	8	One myosin If, four myosin IV, one myosin XIV, two orphan genes [26].
kinesin	30	Members of eight out of 14 families [27]. Twelve orphan or unresolved genes.

### Abundant simple-sequence repeats in the *L. sinaloensis* nuclear genome

We conducted a detailed annotation of repetitive elements in the *L. sinaloensis* genome, including transposable elements (TEs), simple-sequence repeats (SSRs), satellites, etc. The initial repeat library contained 120 sequences. After removing redundancies, merging split elements, and removing sequences with signatures of native genes, this number was reduced to 33 consensus sequences in the final library. Both class I and class II TEs were present in the genome [30], and all repeat families could be assigned to either of the classes. However, apart from having terminal inverted repeats (TIRs), some class II families (*n* = 10) lacked known signatures and could thus not be further classified. The total repeat content in the *L. sinaloensis* genome, including SSRs and interspersed repeats such as TEs, was 10.1%, which is lower than in other rhizarian genomes (Table 1).

**Fig. 2:**
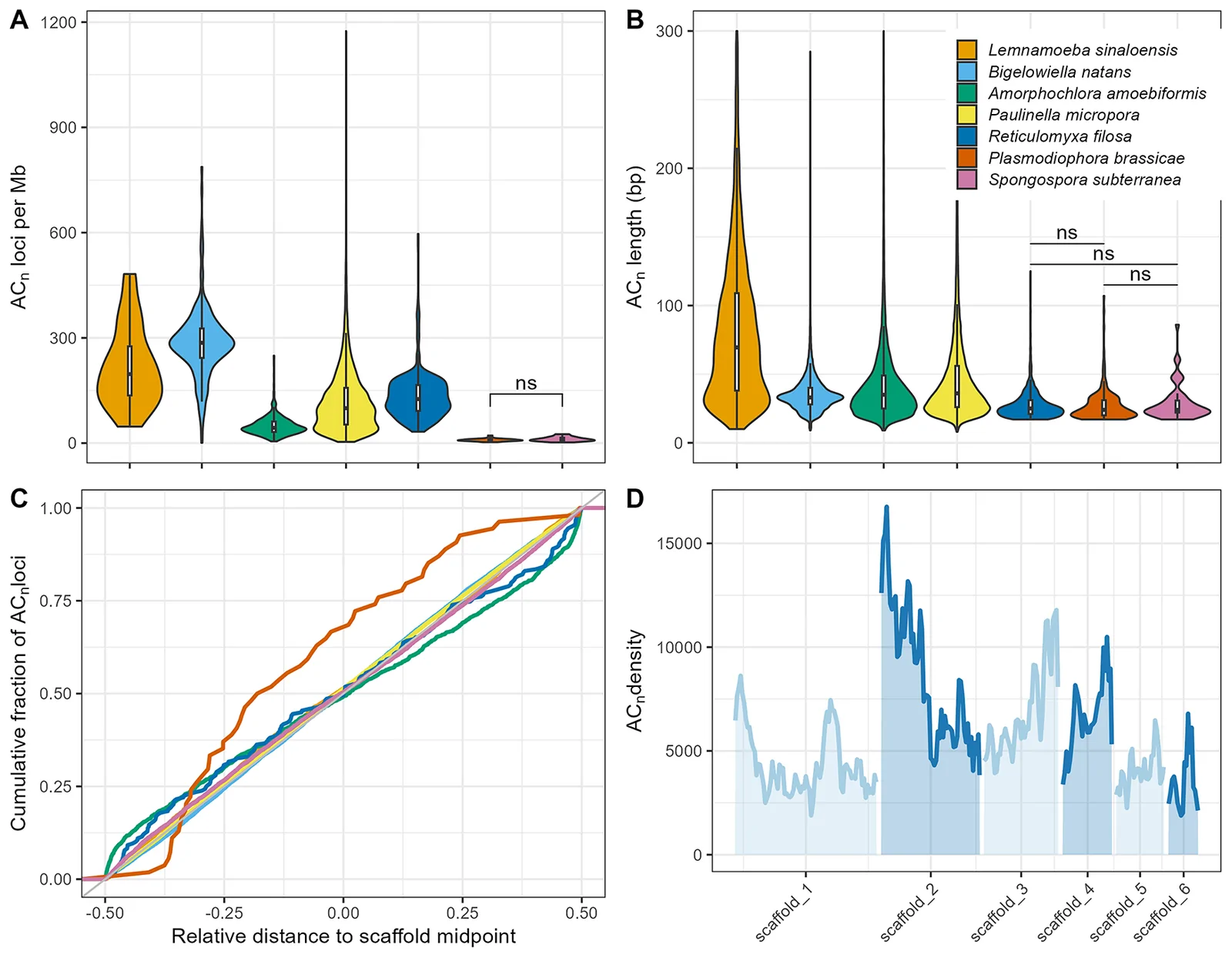
Simple-sequence repeats of the AC:GT_n_ (AC_n_) dinucleotide in rhizarian genomes. **A** Number of AC_n_ loci per Mb. Each genome was divided into bins of maximum 1 Mb in length (shorter scaffolds were used in full; minimum length 5 kb). Data points show the number of AC_n_ loci per bin, normalized for the bin size. **B** Length of all AC_n_ loci in each genome. **C** Cumulative fraction of AC_n_ loci across scaffolds, showing their distribution relative to the scaffold midpoint. Scaffolds were normalized for length. **D** Density of AC_n_ repeats across the *L. sinaloensis* genome in 300 kb windows with 200 kb overlaps. Only the six longest scaffolds are shown. The legend in the top right corner is common for panels A-C. In A and B, pairwise Wilcoxon rank-sum tests were used to compare all genomes against each other. For plotting reasons, only non-significant (ns) results are shown (*p* > 0.05), i.e. all other comparisons are significant (*p* < 0.05)

While the TE content in the *L. sinaloensis* genome is relatively low, the SSR content is strikingly high. In particular, the AC:GT_n_ dinucleotide stands out by covering almost 2% of the total genome size (from hereon simply called AC_n_ repeats Fig. 2). Comparative analysis revealed that this SSR was also very common in the genome of *B. natans*, and to a lesser extent in *R. filosa* and *Pa. micropora*, while being nearly absent in *Pl. brassicae* and *S. subterranea* (Fig. 2A). In *L. sinaloensis*, AC_n_ repeats had a higher number of repetitive units than in other rhizarian genomes (Fig. 2B). In contrast to the previously published short-read assembly [9], the AC_n_ loci in the current assembly were not restricted to contig termini but instead distributed across the scaffolds with some variation in their local density. This feature was also seen in the *B. natans* and *Pa. micropora* assemblies, while *R. filosa* and *Pl. brassicae* showed a trend towards a higher frequency of loci towards scaffold ends (Fig. 2C). In *S. subterranea*, this trend was reversed, with the scaffold centers showing enrichment of AC_n_ repeats (Fig. 2C). Across the *L. sinaloensis* genome, AC_n_ repeats varied in their density, with the second longest contig showing the highest overall density (“scaffold_2”, Fig. 2D). PCR confirmed the presence of all tested AC_n_ loci in *L. sinaloensis*, where lengths of the products corresponded well to the expected lengths from the genome sequence (Fig. S5; Table S3).

### The *L. sinaloensis* gen. nov. et sp. nov. mitogenome is most similar to Retaria

**Fig. 3:**
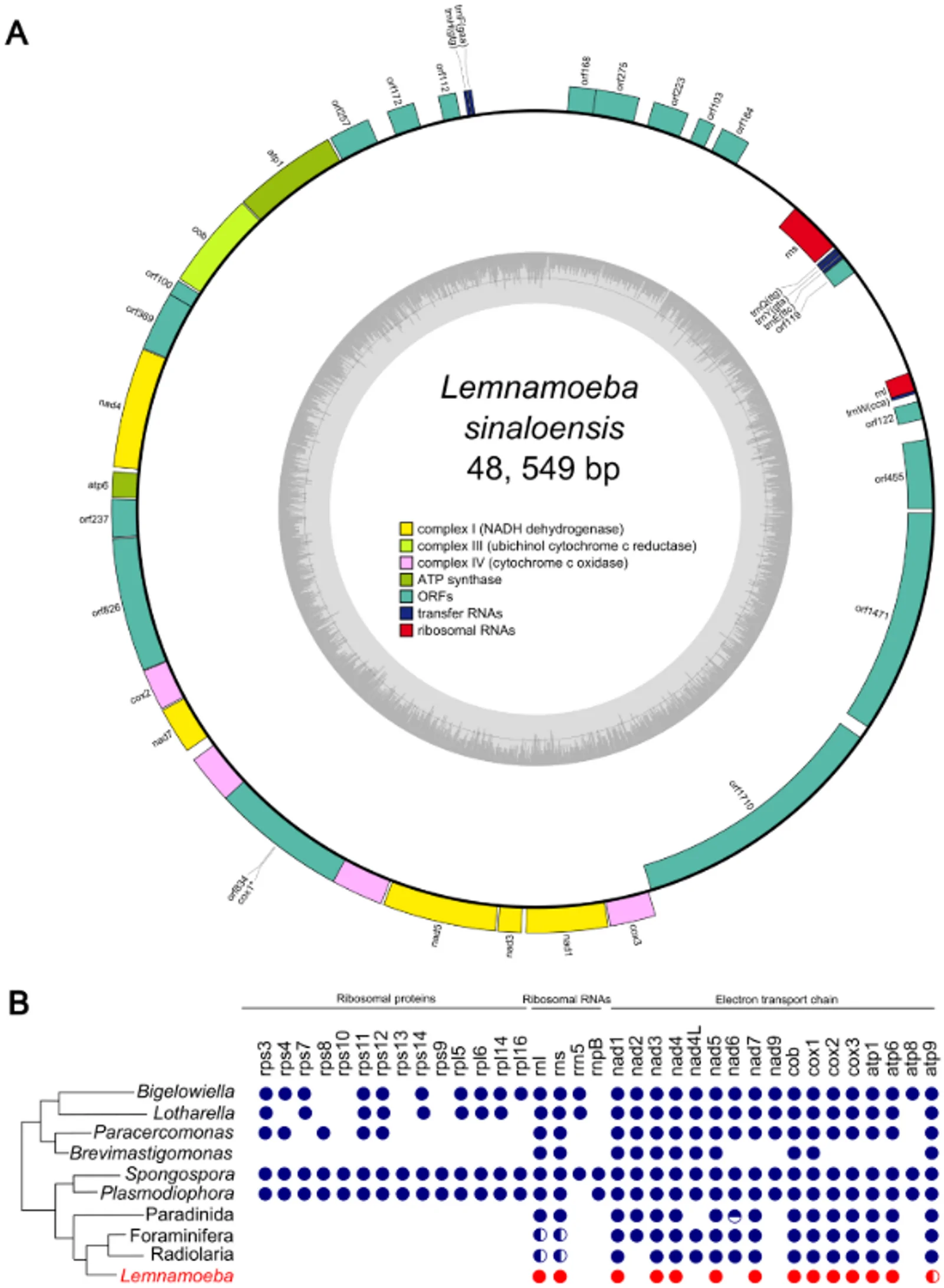
Analysis of the mitochondrial genome of *L. sinaloensis*. **A**. The map of the mitochondrial genome with genes colour coded as to their function, generated using OGDRAW. **B**. Comparison of presence (circle) / absence (no circle) / presence in fragmented form (vertically half-filled circle) / presence in some members and absence in the others (horizontally half-filled circle) of genes in mitochondrial genomes of rhizarians (adapted from [32]).

The full mitochondrial genome of *L. sinaloensis*, extracted from the PacBio contigs, mapped as a circular contig of 48,549 bp with a G + C content of 30.2%, containing 11 protein-coding genes and two ribosomal genes (*rnl*, *rns*) (Fig. 3A). We also identified 18 predicted ORFs, none with detectable homology in NCBI except orf275, which showed some distant hits to proteins from animals (26.28% identical to hypothetical protein BgiBS90_018635 from *Biomphalaria glabrata* snail, E-value 9e-08 and 16.23% identical to hypothetical protein JTE90_004760 from *Oedothorax gibbosus* spider, E-value 8e-07 - NCBI BLASTp search in January 2026). The *Cox1* gene contained a putative retrotransposon (orf834). Using InterProScan search we observed that orf119 and orf122 display significant homology to the *atp9* gene (Table S4), which may be fragmented and very divergent in *L. sinaloensis*. Orf100, orf184, orf223, orf369 and orf826 were predicted to have transmembrane helices, but no functional domains. Orf1710 contained a P-loop with a nucleoside triphosphate hydrolase domain and was predicted to have a DNA/RNA polymerase domain. All other ORFs returned no hits (Table S4).

The similarity of the *L. sinaloensis* mitogenome gene content with that of retarians is noticeable (Fig. 3B). For example, the electron transport chain (ETC) gene set is reduced with *nad9* and *atp8* missing in all representatives, *nad2* gene also missing in Radiolaria, and *nad6* also missing in Foraminifera. *L. sinaloensis* further lacks *nad4L* similarly to ascetosporeans [31]. The mitogenomes of *L. sinaloensis*, ascetosporeans and retarians lacked all ribosomal protein-coding genes (Fig. 3B). Conversely, the *L. sinaloensis* mitogenome retains 6 tRNAs for 6 different amino acids, which are lacking in Retaria and reduced to just 4 tRNAs in ascetosporeans [31]. While missing in the mitogenome, we detected copies of mitochondrial *nad9*, *rps12* and *rps4* in the nuclear genome (Table S5). *Nad9* and *rps12* were predicted to be targeted to mitochondria and are phylogenetically related to mitogenome-encoded sequences in varied species (Fig. S6, Fig. S7). These genes were likely transferred to the nuclear genome in *L. sinaloensis*. *Rps4* also contains a mitochondrial targeting sequence, but this gene is related to bacteria and thus might represent a case of lateral gene transfer in *L. sinaloensis* (Fig. S8). Other nuclear-encoded homologs to missing genes in the mitogenome lacked mitochondrial targeting information, or were not detected (Table S5).

### *L. sinaloensis* gen. nov. et sp. nov. has no close described relatives

**Fig. 4:**
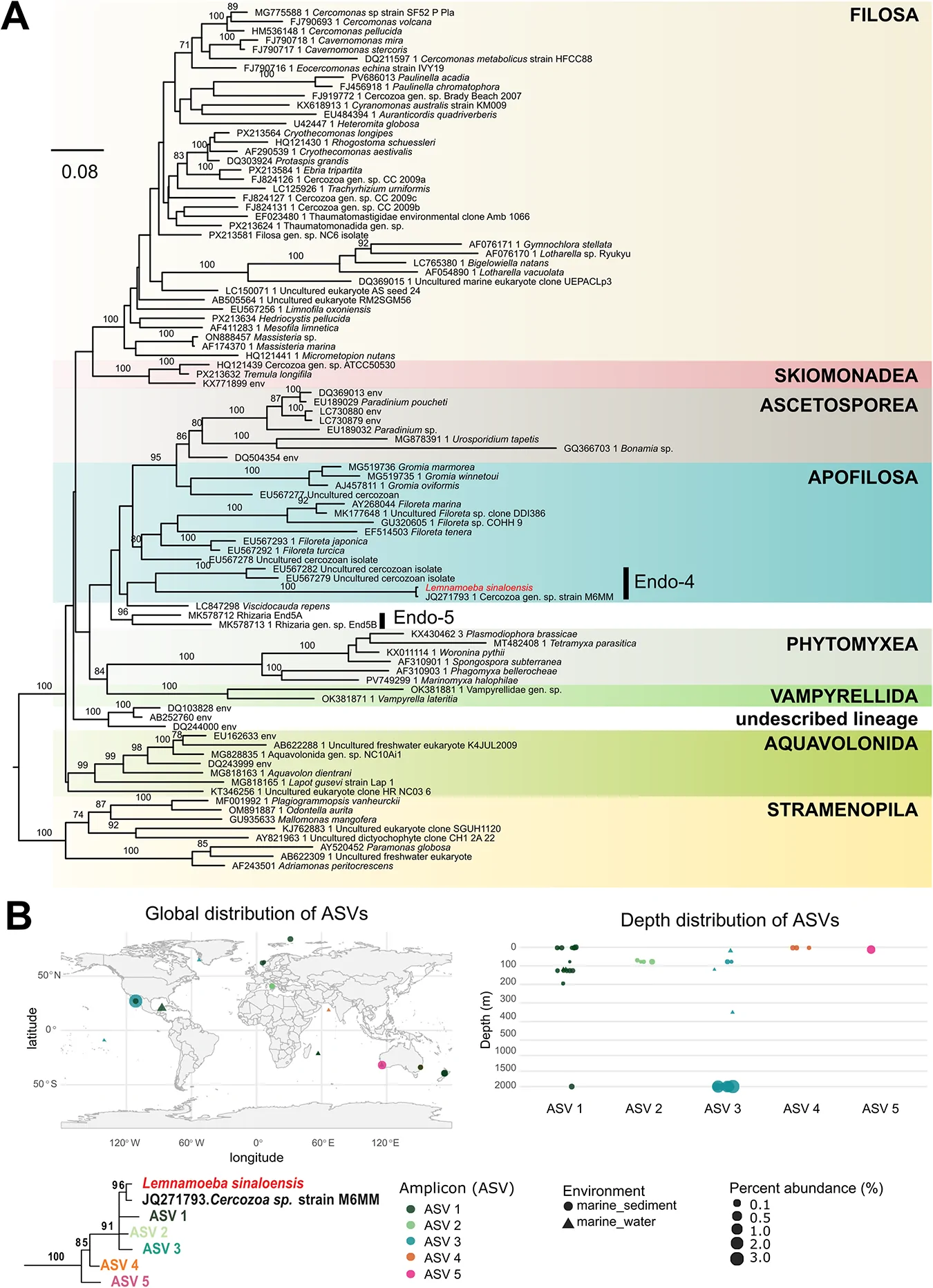
Phylogeny and distribution of *Lemnamoeba sinaloensis* gen. nov. et sp. nov. **A**. Maximum-likelihood phylogeny based on the 18S rDNA sequence of *L. sinaloensis* and homologous sequences. The phylogeny was inferred with IQ-TREE using the best-fit model (BIC) TN+F+I+G4, and numbers on branches indicate standard nonparametric bootstrap support values. Only support values > 70% are shown. A number of Stramenopila species were included as an outgroup for rooting the tree. **B**. Global geographic and depth distribution of EukBank 18S V4 ASVs clustering with *L. sinaloensis* (> 97% identity to the *L. sinaloensis*  18S rDNA sequence). Map points indicate sampling sites where each ASV was detected; for each ASV at a site, a single point shows the maximum relative abundance observed. The depth panel shows ASV occurrence and relative abundance across sampling depths. Colours denote ASV identity, shape indicates environment type, and point size reflects relative abundance (ASV reads/total reads per sample). A zoom-in of the fully supported clade containing *L. sinaloensis* and these ASVs is shown. For the full tree with all twelve identified ASVs, see Fig. S9

While *L. sinaloensis* has been shown to be related to the filose amoeba genera *Filoreta* and *Gromia* in the Apofilosa group in a previous phylogenomic study, this placement lacked a broad diversity context due to a limited taxon sampling where genomic information was available [9]. Here, we inferred the phylogenetic placement of *L. sinaloensis* using a more extensive taxon sampling available for the 18S rDNA (Fig. 4A). Our analysis, restricted to sequences > 1000 bp, showed that no available lineage branched close to *L. sinaloensis*. The only exception was a sequence obtained from the same amoeba culture upon its original isolation (AC: JQ271793; 99.7% similarity), confirming that our newly generated genome originates from the same organism. The closest described species corresponded to the recently described amoeboflagellate *Viscidocauda repens*, although sequence similarity was low (84.5%; AC: LC847298) and the relationship not supported. *L. sinaloensis* was most closely related to environmental sequences previously annotated as Endo-4 environmental lineage [33], but this grouping again lacked support. Generally, our 18S rDNA analysis recovered a paraphyletic Apofilosa, with *Gromia* branching with Ascetosporea and *Filoreta* sister to that group. Given the lack of closely related described species to *L. sinaloensis*, we here propose to erect a new genus for this amoeba with *Lemnamoeba sinaloensis* gen. nov. et sp. nov. designated as the type species.

### *L. sinaloensis* gen. nov. et sp. nov. shows a patchy but global marine distribution

To evaluate the diversity and geographical distribution of *L. sinaloensis* when also including shorter metabarcoding data, we queried the EukBank 18S‑V4 dataset [34] and recovered 12 ASVs ≥ 97% similar to *L. sinaloensis*. Phylogenetic placement grouped only five ASVs with *L. sinaloensis* (Fig. S9). One ASV showed > 99% similarity, whereas the remaining four were 97.47–98.99%. All five occurred exclusively in marine samples and were cosmopolitan—from Arctic to subtropical waters—but detected in only a few samples (50/12672; Fig. 4B; Table S6). They were most frequent in sediments, with occasional, lower‑abundance detections in the water column (Fig. 4B). Notably, ASV3 reached > 3% relative abundance in deep (~ 2,000 m) sediments collected off the coast of Mexico.

## Taxonomy

SAR supergroup Burki et al. 2007, emend. Adl et al. 2012.

● Rhizaria Cavalier-Smith 2002.

●● Apofilosa Sierra et al. 2016, emend. Burki & Hiltunen Thorén.

Node-based definition: The least inclusive clade containing *Filoreta tenera* (Tekle, Grant, Cole, Nerad, Anderson, Patterson & Katz 2007) Bass & Cavalier-Smith 2009; *Gromia sphaerica* Gooday, Bowser, Bett & Smith 2000, and *Lemnamoeba sinaloensis* sp. nov. but not *Marteilia pararefringens* Bass, Stentiford and Kerr 2017, *Elphidium margaritaceum* Cushman 1930, or *Amphilonche elongata* (Müller 1858) Haeckel 1862.

Remarks: The name was informally introduced for “clade *Gromia* + *Filoreta*” [8]. Apofilosa appeared in some subsequent sources, but here is formally described for the first time.

### *Lemnamoeba* Holzer & Pecková gen. nov.

#### Diagnosis

Marine lobose amoebae lacking a test. Differs from *Gromia* Gooday et al. 2000 by the complete absence of an organic test or shell with a distinct oral aperture. Differs from *Filoreta* [35] by existing predominantly as individual, small, non-plasmodial amoeboid cells moving via lobopodia rather than forming extensive reticulate, plasmodial networks. Differs from other naked Rhizaria by the presence of a unique cytoplasmic structure composed of densely packed, ribbon-like parallel tubules.

#### Description

Marine lobose amoebae without a test, rounded but often of variable shape. In crawling and attached stages frequent formation of lobopodia and separation in a central refractile granuloplasm and clear outer hyaloplasm. Occasional filiform adhesive uroidal structures and of variable length. Cytoplasm contains a characteristic but unidentified structure of packed tubules.

#### Etymology

From Greek lemniskos (λημνίσκος, “ribbon” or “fillet”) + amoeba, referring to the packed tubules of ribbon-shaped appearance which were commonly found in the cytoplasm.

#### Remarks

The genus Lemnamoeba is currently monotypic, containing only the type species *Lemnamoeba sinaloensis* sp. nov.

#### ZooBank LSID

 B0CDE136-8887-42E3-AB15-F87778173AB8

### *Lemnamoeba sinaloensis* Holzer & Pecková sp. nov.

#### Diagnosis

Marine lobose amoeba with cell body diameter of 5–18 μm; cells mostly rounded when unattached to oblong and variously shaped when crawling; frequent blunt lobopodia, uninucleate or occasionally binucleate; mitochondria possess tubular cristae; cytoplasm with 1–2 dense bodies per cell and packed parallel tubules.

#### Description

Trophozoite cell bodies 5–18 μm, variable in shape from rounded to oblong or irregularly shaped (Fig. 1A, Additional File 1). Amoeboid movement via lobopodia (Fig. 1B); occasional adhesive uroidal structures filiform and 1–6× body length (Fig. 1B, C); locomotive forms 7.2–26.8 μm long (*n* = 48); usually one, sometimes two nuclei per cell, nuclei of variable shape, often elongate and previously reported lobed [36]; cytoplasm with ribosomes, numerous vesicles, vacuoles and 1–2 dense bodies per cell (Fig. 1F), food vacuoles frequently containing rod-shaped bacteria (Fig. 1G), elongate (*Flammeovirga* sp.) and ovoid (*Pseudorhodobacter* sp.), identified by genome sequencing, co-isolated and abundant in culture medium (Fig. 1A, B,D); late-stage food vacuoles commonly containing membrane-within-membrane remnants (Fig. 1H); mitochondria with tubular cristae (Fig. 1F); common unidentified structure of packed tubules in parallel arrangement (Fig. 1E, F). The description matches morphological details observed in the original isolate of the same amoeba strain, named ‘M6MM’ under ‘strains incertae sedis, group 05’ [36], p. 345).

#### Etymology

sinaloensis (from Sinaloa, Mexican state at the Gulf of California) - after the type location.

#### Type locality

Isolated from the finescale triggerfish, Balistes polylepis Steindachner, collected off Mazatlán, Sinaloa, Mexico.

#### Type material (hapantotype)

Two glass slides with fixed individuals (85% glycerol, 15% phosphate-buffered saline) of the type strain deposited in the Protist Collection of the Institute of Parasitology, Biology Centre of the Academy of Sciences of the Czech Republic with the specific reference number IPCAS_89. Associated deposited materials: Extracted DNA and cryopreserved amoebae (stored since 2000) for culture on MY75S agar overlaid with seawater. 

#### DNA sequence data

BioProject PRJNA1345024; raw sequencing data can be accessed at the NCBI Sequencing Read Archive (SRX30991339) and annotated genome assembly in the NCBI genome database (GCA_040357565) . SSU rDNA sequence deposited under JQ271793 [36].

#### ZooBank LSID

84C143CA-F618-4429-9024-72A01E7729C8

## Discussion

In this work, we described the lobose amoeba *L. sinaloensis* gen. nov. et sp. nov., and assembled its genome to a high level of contiguity. Our phylogenetic analysis loosely placed *L. sinaloensis* with apofilosan lineages. Apofilosa was originally proposed to include the filose amoebae *Gromia* and *Filoreta* [8]. Our tree did not recover a monophyletic Apofilosa, but the support in general for the branching of apofilosans and related undescribed lineages was low (Fig. 4A). However, the relationship of *L. sinaloensis* with *Gromia* and *Filoreta* previously received moderate to high support in a phylogenomic analysis [9], indicating that this group is genuine. Based on the large phylogenetic distances to the closest described species, we propose to erect the new genus *Lemnamoeba*. Apart from being amoeboid, the few described species in Apofilosa lack shared diagnostic morphology. *Gromia* is a giant shelled filose amoeba [37], *Filoreta* forms flattened multinucleate plasmodia [38], and *Lemnamoeba* comprises small, motile, amoeboid, uninucleate cells. The tentative placement of *Viscidocauda* within Apofilosa requires phylogenomic testing, as our 18S rDNA tree is inconclusive. If confirmed, *Viscidocauda* flagellate phenotype would further increase the heterogeneity of the group [39]. This morphological patchiness likely reflects uncharacterized diversity, as several related lineages (e.g., Endo‑4, Endo‑5) are only known from environmental sequences [33].

We performed cytological and genomic characterisation of *L. sinaloensis*. The cytology showed the presence of six well-defined chromosomes which correlates with our genomic findings. The structural and functional annotation of the *L. sinaloensis* genome identified several unusual features when compared to other eukaryotes. First, we were unable to detect canonical telomeres, which are usually easy to identify with long-read sequencing [40]. The cause of this may be technical, such as degradation of the DNA by exonuclease activity during or after extraction. Still, we identified one G-rich repetitive motif (TAA[G]_n_) at some of the contig ends in *L. sinaloensis.* However, it also occurred within contigs, indicating that this motif was not specifically associated with the telomeres. In *Pl. brassicae*, the telomere repeat motif is non-canonical (T_n_AG_n_), but restricted to chromosome ends [19]. In *B. natans*, the canonical repeats are found, and in other Rhizaria, the telomeres are undescribed.

The *L. sinaloensis* genome carries an average of thirteen introns per gene. Across species, the average intron number is typically correlated with genome size, with between four and seven introns per gene in multicellular eukaryotes, and fewer in unicellular ones [41]. Recent reports from the filosan *A. amoebiformis* and green alga *Chlamydomonas* sp. have shown that unicellular eukaryotes may also carry many introns [22, 42], but the high number in *L. sinaloensis* stands out given its tiny genome size. The high number of introns also stands in contrast with genomes in the related parasitic group Ascetosporea, where intron numbers and sizes have been drastically reduced [9, 15, 43]. High intron numbers have been related to insertions of a specific type of TE termed Introners [44], but no such signal could be detected in the *L. sinaloensis* introns, suggesting that some other mechanism might be responsible for intron introduction and retention in this organism.

Within Rhizaria, it has been suggested that retarians have a high diversity of tubulins, supporting stiff reticulopodia, while filosans rely more on actins to build their cell structure and motility by filopodia [26]. This observation was supported by the finding that the genome of *R. filosa* (Retaria) encodes a higher number of tubulin than actin genes [18]. In *L. sinaloensis*, we found few tubulin genes while recovering many actin genes with high sequence similarity, suggesting recent duplications. In combination with the proposed phylogenetic placement of *L. sinaloensis* closer to Retaria than Filosa based on phylogenomics [9], these results suggest that cytoskeleton gene content in Rhizaria is dynamic and may evolve in concordance with the motility mode of the organism. Similarly as in *R. filosa*, we identified a high number of kinesin genes, supporting that this family of motor proteins is important in Rhizaria [18].

An interesting genomic property of *L. sinaloensis* is the large amount of SSRs, particularly of the AC_n_ type (Fig. 2). Compared to model organisms, many rhizarian genomes are much richer in AC_n_ SSRs, carrying up to an order of magnitude more loci per Mb [45]. In our comparative analysis of Rhizaria, *B. natans* has the highest density of AC_n_ loci in its genome, followed by *L. sinaloensis*, while the phytomyxeans *Pl. brassicae* and *S. subterranea* are almost devoid of SSRs (Fig. 2A). The number of repetitive units (i.e., length per locus) is by a clear margin the highest in *L. sinaloensis* (Fig. 2B). In the assembly of *R. filosa*, while the amount of AC_n_ SSRs is lower than in *L. sinaloensis* and *B. natans*, it was noted that many short, repetitive contigs were filtered out during the assembly process, so it is possible that this species also carries a high amount of SSRs [18]. The reason multiple rhizarian species have large amounts of SSRs in their genomes is unknown. Simple-sequence repeats mutate easily due to polymerase slippage causing insertions or deletions of the repetitive unit [46]. The DNA mismatch repair pathway is used to repair replication errors such as polymerase slippage [47]. We found candidates for the mismatch repair genes *MSH2*, *MSH3*, and *MSH6* in the *L. sinaloensis* genome (Table S2), indicating that the pathway is intact in this organism. Simple-sequence repeats such as AC_n_ are also known to promote formation of Z-DNA from the canonical B-DNA form [48, 49]. Notably, in the ciliate *Stylonychia mytilus*, Z-DNA has been found in the transcriptionally active macronuclei, but not in the silent micronuclei, suggesting a regulatory role [50]. The high abundance of AC_n_ repeats in the *L. sinaloensis* genome presents an intriguing avenue for further research into Z-DNA formation in a protist.

In addition to the nuclear genome, we assembled the mitochondrial genome of *L. sinaloensis*. Its gene content is the lowest among known rhizarian mitogenomes with aerobic mitochondria, only slightly exceeding that of the reduced mitochondria-related organelle (MRO) genome of the microaerophilic *Brevimastigomonas motovehiculus*: 11 genes of ETC retained, compared to eight in *Brevimastigomonas* [51] (Fig. 3B). It ranks among the most reduced mitogenomes in eukaryotes, resembling the extremely simplified genomes of animals (e.g., human) and green algae (e.g., *Chlamydomonas*), retaining only an incomplete set of ETC components [52]. Several predicted hypothetical proteins are likely non-functional and possibly unexpressed, similar to the large intergenic regions reported in retarian mitogenomes [32]. The interruption of *cox1* by a transposon suggests either loss of function or RNA-level splicing of the fragments. No synteny was detected with other rhizarian mitogenomes, indicating extensive rearrangements as previously observed [53]. Like in *B. motovehiculus* and retarians, *L. sinaloensis* lacks all ribosomal protein genes, implying complete loss or transfer to the nucleus. Supporting this second option, we identified a nuclear-encoded mitochondrial copy of *rps12* with targeting prediction, which could have been transferred from the mitogenome. We propose a similar transfer for *nad9*. The shared absence of ETC genes further supports similarity with retarian mitogenomes, consistent with the phylogenetic placement of Apofilosa as sister to Retaria [9]. Alternatively, if Apofilosa and Endomyxa are closer to Cercozoa than to Retaria [5], the mitogenome of *L. sinaloensis* must have converged independently on a retarian-like organization.

In conclusion, our work adds a new genus to the group Apofilosa that previously consisted only of the genera *Gromia* and *Filoreta* and some environmental lineages [8], thus adding important information about this poorly known group of Rhizaria. Given the large diversity and ecological importance of Rhizaria, it is striking that the number of high-quality genome sequences to date is so low. Using long-read sequencing and relatively simple assembly pipeline, we here show that rhizarian genomes are attainable, at least when the organism is in culture and the genome is small. Going forward, sequencing efforts should continue to explore undersampled groups in Rhizaria, which will fill in additional knowledge gaps of eukaryote genome evolution.

## Materials and methods

### Strain isolation & culturing conditions

Isolation of *L. sinaloensis* M6MM has been described previously [9]. Briefly, the amoeba was isolated from the gills of finescale triggerfish, *Balistes polylepis*, sampled in Mazatlan, Mexico, in July 2000. Gill clips were placed on malt and yeast extract seawater agar (MY75S in the Culture Collection of Algae and Protozoa, SAMS Ltd, Oban, UK). Isolated amoebae were subcultured on the same medium. Subculturing was performed by transferring amoebae on agar slices to fresh plates. Plates were incubated at 20 °C. Cryopreservation of amoebae was done in seawater containing 10% DMSO and stored in liquid nitrogen at the culture collection of the Institute of Parasitology, Biology Centre of Czech Academy of Sciences.

### Microscopic observation

For microscopical preparations amoebae were washed from agar plates using aseptic seawater, concentrated by centrifugation (2000 × g, 8 min) and washed twice with aseptic seawater. Light microscopic observations were carried out in hanging drop preparations, using translucent light and differential interference contrast (DIC) optics on an Olympus BX51 microscope (Tokyo, Japan). Nuclei in live amoebae were stained using NucBlue™ Live ReadyProbes™ Reagent (Hoechst 33342). Digital images were taken at a 400× and 1000× magnification using an Olympus DP70 camera (Tokyo, Japan). Measurements were obtained from digital images of 20 live trophozoites using ImageJ 1.53e software [54]. Digital videos were taken at 1000x magnification using Lumenera Infinity 1 camera, the scale was acquired with a transmitted light object micrometer 1 mm/100 (Olympus OB-M) and pasted to the videos using FiJi ImageJ 1.54p software. For transmission electron microscopy (TEM) washed and pelleted amoebae were fixed in 2.5% glutaraldehyde in 0.1 M cacodylate buffer, the pellet immobilized in 10% gelatine, post-fixed with 1% osmium tetroxide, and dehydrated in an ascending alcohol series. Samples were then dehydrated, transferred and embedded in 60% epoxypropane/40% epon araldite solution. Ultrathin sections were cut, mounted on grids and contrasted with uranyl acetate and lead citrate. Grids were examined using a JEM 100B transmission electron microscope (JEOL Ltd).

### Cytogenetic analysis

To arrest nuclear division at metaphase, the central part of the amoebae culture containing older, non-dividing cells was cut out to increase the proportion of dividing cells [55]. The agar plates containing amoebae cultures were overlaid with 0.05% colchicine in aseptic seawater and incubated for 24 h. The cells were then washed from the agar with aseptic seawater, centrifuged, and the resulting pellet was subjected to hypotonic treatment with 25% aseptic seawater in distilled water for 10 min. After removal of the hypotonic solution, the cells were fixed in for at least 5 min. Amoebae were spread onto clean slides in 60% acetic acid and air-dried. The dried slides were stained and mounted with a histology mounting medium, Fluoroshield with DAPI (Sigma-Aldrich), and the preparations were observed and photographed using an Olympus BX51 microscope equipped with an Olympus DP70 camera. The chromosomes were measured using ImageJ 1.54 g software.

### DNA extraction, library preparation & sequencing

To extract DNA, we washed amoebae off the agar plates with seawater followed by brief centrifugation. Pelleted amoebae were dissolved in TNES buffer (4 M urea; 10 mM TrisHCl, pH 7.5; 120 mM NaCl; 10 mM EDTA; 5% SDS), and DNA was extracted using a standard phenol–chloroform extraction method. At Uppsala Genome Center (UGC), genome sequencing libraries were prepared from extracted DNA using the HiFi SMRTbell Express Template Prep Kit 2.0 + Enz Clean Up (large insert) (Pacific Biosciences) and sequenced using the PacBio Sequel II platform on one HiFi-SMRT cell. The dataset was supplemented with previously generated short-read genome and transcriptome sequencing data [9].

### Genome assembly

PacBio HiFi reads were assembled using HiFiasm v0.19.6 at UGC. The same reads were mapped back to this assembly using Minimap2 with the map-hifi preset [56]. The mitochondrial genome was identified as a single contig by having lower GC content than the majority of sequences, carrying mitochondrial genes, and having overlapping ends, indicating circularity. As the data was derived from a culture containing both *L. sinaloensis* and bacteria, we expected contigs of bacterial origin to be part of the assembly. To exclude bacterial DNA, contigs with a GC content lower than 0.4 or higher than 0.6 were removed. To improve contiguity of remaining contigs, some additional steps were taken as follows. HiFi reads mapping to the contigs with a GC content between 0.4 and 0.6 were extracted and re-assembled using both Canu v. 2.2 [57] and Miniasm v. 0.3 [58]. Based on overall contiguity, the Canu assembly was selected as a backbone for further curation, and to this assembly, our previously generated dataset of 10X Chromium linked reads (NCBI SRA: SRX22918778) was mapped using BWA v. 0.7.17 with the ‘mem’ algorithm [59]. Based on the linkage information from this dataset, ARBitR v. 0.2 [60] was used to find associations between contig ends. Linkage of Canu-generated contigs was determined based on three lines of evidence. First, we required that they were joined by the other two assembly programs, which we investigated by whole-genome alignment of all assemblies against each other using the nucmer module of MUMmer v. 3.23-13 (default parameters) [61]. Second, we required the contigs to share a significant proportion of linked-read barcodes, as determined by ARBitR [60]. Finally, we required the ends of the putatively linked contigs to overlap, thus not allowing any scaffold gaps in the final assembly. Pairs of contigs meeting these criteria were merged using a custom script (https://github.com/markhilt/genome_analysis_tools/blob/master/overlap_scaffolding.py*).* Resulting merged contigs were polished for remnant sequencing errors by remapping HiFi (Minimap2 with same settings as above) and 10X Chromium reads mapped with EMA v. 0.6.2 [62], before running HyPo [63].

To validate that the genome assembly was a good representation of the nuclear genome, we used a combination of KAT v. 2.4.2 and GenomeScope2 v. 2.0 for a k-mer based genome size estimation [64, 65]. We used the PacBio HiFi reads for this assessment. Since the dataset contained reads of bacterial origin that would skew the results, we started by filtering the reads based on k-mer coverage and GC content with the ‘filter’ module of KAT (k = 31, k-mer coverage: 50–600, GC count: 5–17). The resulting read set (containing 610,517 out of 2,690,294 PacBio HiFi reads; 23%) was used as input for GenomeScope2, with k-mer counting and histogram construction by jellyfish v. 2.3.1 [66].

### Annotation of repetitive elements

The *L. sinaloensis* genome assembly was subjected to annotation of repetitive elements. RepeatModeler v. 2.0.2 [67] was used to mine the genome for repetitive sequences, resulting in 120 putative families of transposable elements (TEs) and other repeats. The library of repetitive sequences went through a manual curation pipeline as follows. From the initial repeat library, the consensus sequence for each repeat family was aligned back to the genome with BLASTn. Hits were extracted (including flanks of 1000–5000 bp, depending on the TE family), and the extracted sequences (putative TE copies) were aligned using MAFFT. Boundaries of TE copies aligned this way were examined for structural signatures in the form of target site duplications (TSDs), terminal inverted repeats (TIRs), long-terminal repeats (LTRs), and polyA tails of LINEs. Boundaries of RepeatModeler TE families were extended where needed, and new consensuses were created using majority-rule consensus within Geneious v. 10.2.6 (www.geneious.com*).* Resulting sequences were searched for conserved domains using the Conserved Domain Database online (CDD; https://www.ncbi.nlm.nih.gov/Structure/cdd/wrpsb.cgi*).* Taking all structural and domain information into account, new TE family consensus sequences were classified. Putative repeat families without clear boundaries and where no TE protein domains or structural signatures could be found were excluded from the repeat library. Many such sequences carried protein domains not usually found in TEs, instead likely representing multi-copy genes. The resulting library of TEs was used to annotate repeats with RepeatMasker v. 4.1.5 [68], which also identified SSRs in the *L. sinaloensis* genome.

### Annotation of genes in the nuclear genome

Gene sequences were annotated in the new genome assembly as described for the previous *L. sinaloensis* genome version [9]. The Funannotate v. 1.8.9 [69] pipeline was used with the newly generated repeat library to mask repeats. Training of ab initio gene prediction software was done using the Funannotate “train” module, providing our previously generated transcriptome sequencing data (SRA: SRX22918777) for support. After training, the modules “predict”, “update”, “iprscan”, “phobius”, and “annotate” were run in that order, to generate functionally annotated gene models of the genome. Completeness statistics of the genome assembly and annotation were collected with BUSCO v. 6.0.0 [70] in “genome” and “protein” modes, respectively. Since no ortholog database for Rhizaria exists in OrthoDB, we used the “alveolata_odb12” BUSCO lineage. Based on the functional annotation, cytoskeleton and motor protein genes were extracted. Kinesin homologs were predicted based on carrying the motor domain (IPR036961 or IPR001752), and excluding myosin genes. For kinesin family membership inference, reference sequences were collected (excluding the sequences AAA35056 and AAA28653 due to their difficulty to align [27]), and aligned with putative kinesin protein sequences from *L. sinaloensis* using MAFFT v7.526 with the E-INS-I algorithm. A tree was computed with IQ-TREE v. 3.0.1 with the LG+F+I+R6 model. Family assignment was determined based on inclusion of *L. sinaloensis* sequences in a supported (UFBS > 95) clade with reference sequences. Nucleotide sequences of the eleven putative actin homologs were used as queries to search for similar sequences in the nt database of GenBank (BLASTn search performed 2026-08-12). Since actin is a highly conserved gene, the e-value of all hits was close to 0. The 100 hits with highest percent identity were retained for each actin homolog, compiled into a list where each unique accession was kept once (173 sequences remaining), aligned with the MAFFT E-INS-I algorithm, and a tree was computed with IQ-TREE. Genes of the mismatch repair pathway were identified by BLASTp (e-value < 1E-30) using queries from the well-annotated *Dictyostelium discoideum* (UniProt: Q553L4, Q1ZXH0, Q55GU9), and the functions of candidate genes were predicted by online searches at InterPro and CDD.

### Annotation of the mitogenome

The contig corresponding to the mitogenome was extracted to a separate fasta file and annotated using the MFannot webserver [71] using #4 genetic code table (The Mold, Protozoan, and Coelenterate Mitochondrial Code and the Mycoplasma/Spiroplasma Code), the obtained .sqn file was converted to GenBank format using asn2gb (https://ftp.ncbi.nlm.nih.gov/asn1-converters/by_program/asn2gb/*)* GenBank tool. The genome map was generated using OGDRAW39 v. 1.3.1 [72]. For protein domain homology survey of ORFs we used InterProScan proteins [73] with default parameters.

### Endosymbiotic gene transfer

Endosymbiotic gene transfer (EGT) of genes from the mitochondrial to the nuclear genome was investigated as follows. The list of genes that could not be identified in the mitogenome (Fig. 3B) was searched for in the nuclear genome by BLAST. Protein models of these genes from *Pl. brassicae* were collected (Table S5) and homologs were searched for in the *L. sinaloensis* protein models using BLASTp (e-value < 1e-1, query lengths 98–490 aa). Genes missing from the annotation and/or pseudogenes were searched for in the nuclear genome with tBLASTn (e-value < 1e-1). Protein domains of hits were annotated with CDD-search online (https://www.ncbi.nlm.nih.gov/Structure/cdd/wrpsb.cgi*)* to confirm gene identity. For protein models with the correct identity, the localization was predicted using DeepLoc v. 2.1 (https://services.healthtech.dtu.dk/services/DeepLoc-2.1*).* To further confirm the mitochondrial origin of these genes we built phylogenetic trees using the protein models for all the genes that were predicted to carry a targeting signal (*rps4*,* rps12*, and *nad9*). These genes were used as queries for BLASTp searches of the NCBI nr database (e-value < 1e-11, 1e-34, 1e-61, for RPS4, RPS12, and NAD9, respectively, search performed 2026-09-25). Sequences for the top 100 hits per protein model were extracted, aligned (MAFFT v. 7.526; L-INS-I algorithm) and trees were computed with IQ-TREE v. 3.0.1, testing for the best-fit model of sequence evolution (LG+I+G4 for NAD9 and RPS4, LG+G4 for RPS12), and 1000 ultrafast bootstrap replicates.

### Distribution analysis

The EukBank dataset, comprising the 18S V4 rDNA dataset and including 12,672 globally distributed metabarcoding samples [34], was used for biogeographical analysis. The 18S rDNA sequence was extracted from the *L. sinaloensis* genome and used as a query sequence in BLASTn analysis against the EukBank database (97% sequence identity threshold), and the closest hits were used in downstream phylogenetic analysis (Fig. S9, Table S6). Metadata analysis was focused on the amplicon sequencing variants (ASVs) clustering together with *L. sinaloensis* in the 18S rDNA phylogeny (Fig. S9, Table S6). EukBank files were analysed in RStudio v. 2025.09.2 + 418 using R v. 4.5.2. The EukBank files containing ASV names, metadata, and number of reads were merged to generate a combined file focusing on the *Lemnamoeba*-related ASVs (Table S7). Global biogeographic distribution and depth analysis of the ASVs was conducted in R. The code used for all analyses is provided as an R script (https://github.com/yashpardasani/Lemnamoeba_sinaloensis_biogeography*).*

### Phylogenetic analyses

The phylogenetic placement of *L. sinaloensis* was inferred using a maximum-likelihood approach. The extracted 18S rDNA sequence from the *L. sinaloensis* genome was used as a query. Similar sequences were collected using an online BLASTn search. Initially, 100 homologous sequences were retrieved from the NCBI database (search performed on 25 November 2025) using the highly similar sequences (megablast) algorithm with default parameters. The blast hits included a sequence generated upon initial isolation of the amoeba (JQ271793.1), and we included it in the analysis to confirm that our genome originates from the same organism. The resulting FASTA file was manually supplemented with 18S rDNA sequences from Rhizaria retrieved from previous studies [74] and aligned using MAFFT v. 7 via the online server (https://mafft.cbrc.jp/alignment/server/index.html*)* with the iterative refinement method E-INS-i. After model selection, a maximum-likelihood phylogeny was inferred from the alignment, and branch support was assessed using 1,000 ultrafast bootstrap replicates. The model selection, phylogenetic inferences and bootstrap support were performed using IQ-TREE v. 2.0 [75]. This initial tree was inspected, and redundant sequences or sequences with excessively long branches were manually removed from the FASTA file. The curated FASTA file was realigned using MAFFT v. 7 with the E-INS-i algorithm. TrimAl v1.5.1 [76] was used to trim the multiple sequence alignment using a gap threshold of 0.2. IQ-TREE v. 3.0.1 was used for selecting the best-fit model (TN+F+I+G4 according to BIC) and reconstructing the best tree using 100 bootstrap replicates.

The phylogenetic placement of ASVs retrieved from the EukBank 18S V4 rDNA database was inferred by adding the ASV sequences to the curated FASTA file used to generate the final *L. sinaloensis* phylogeny. The combined dataset was realigned using MAFFT v. 7 with the E-INS-i algorithm. The nucleotide substitution model, the maximum-likelihood phylogenetic inference and the branch support was assessed using 1,000 ultrafast bootstrap replicates with IQ-TREE.

### Molecular confirmation of simple-sequence repeat loci

To verify that SSRs were not artifactually introduced during library preparation or genome sequencing, we performed molecular amplification of ten randomly selected loci of the AC:TG dinucleotide repeat by PCR. Primers were designed in unique flanking sequence around the SSRs using Primer3 [77] (Table S3). PCR was performed using the EconoTaq PLUS Master Mix Kit following the manufacturer’s instructions and the same DNA isolate used for the genome sequencing. Each 25 µL reaction contained 12.5 µL EconoTaq PLUS Master Mix, 2.5 µL of each primer (10 µM), and 6.5 µL Milli-Q water. The thermocycling parameters were as follows: initial denaturation at 94 °C for 2 min; 30 cycles of 94 °C for 30 s, annealing at 63 °C for 30 s, and extension at 72 °C for 1 min; followed by a final extension at 72 °C for 10 min and a hold at 4 °C. PCR products were visualized on a 1.5% (w/v) agarose gel prepared in 1×TAE buffer and electrophoresed for 1 h at 60 V. The GeneRuler 100 bp DNA Ladder was used as a molecular size standard to estimate PCR product band lengths.

### Comparative analyses of gene and repeat content

We performed comparative analyses with public genome sequences of Rhizaria. We excluded Ascetosporea because of their special genome evolution [9, 43], as well as partial and low-quality rhizarian genome assemblies. The genome sequence files of *B. natans*,* R. filosa*,* Pl. brassicae*,* S. subterranea*,* Pa. micropora*, and *A. amoebiformis* in fasta format were obtained from NCBI GenBank. Where available, gene annotations in gff format were also obtained. To calculate basic genome assembly statistics, we used a custom script (https://github.com/markhilt/genome_analysis_tools/blob/master/n50.py*).* Statistics of genes, introns, etc. were obtained using a combination of GAG v. 2.0.1 [78] and AGAT v. 1.7.0 [79]. Where gene annotations were not publicly available (*S. subterranea*,* Pa. micropora*), the original publications were directly mined for relevant statistics. Repetitive genomic elements were annotated in each genome using the RepeatModeler v. 2.0.4 (including LTRpipeline) and RepeatMasker v. 4.1.5 pipeline [80]. Comparative analyses were performed regarding genome size, gene numbers, intron numbers, and number of loci and repetitive units of SSRs.

## Supplementary information

Below is the link to the electronic supplementary material.

Supplementary Material 1

Supplementary Material 2

Supplementary Material 3

Supplementary Material 4

Supplementary Material 5

## Data Availability

All newly produced data are available on NCBI under the BioProject PRJNA1345024. The annotated *L. sinaloensis* genome is available in the NCBI genome database (GCA_040357565). PacBio sequencing data have been deposited at the NCBI Sequence Read Archive (SRX30991339).

## Abbreviations

TEM: transmission electron microscopy
DIC: differential interference contrast
BUSCO: benchmarking universal single-copy orthologs
TE: transposable element
SSR: simple-sequence repeat
TIR: terminal inverted repeat
ORF: open reading frame.
ETC: electron transport chain
EGT: endosymbiotic gene transfer
ASV: amplicon sequencing variants
SAR: Stramenopila-Alveolata-Rhizaria
MRO: mitochondria-related organelle
