# Supplementary material for: Chromosome-level assembly of Lemnamoeba sinaloensis gen. nov. et sp. nov. (Apofilosa) reveals dynamic genome evolution in Rhizaria
Source: BMC Genomics. 2026 Oct 28;27:933. doi: 10.1186/s12864-026-13502-3 (PMC13746701; doi:10.1186/s12864-026-13502-3)
Supplement: Supplementary file 1 [file 12864_2026_13502_MOESM5_ESM.pdf]

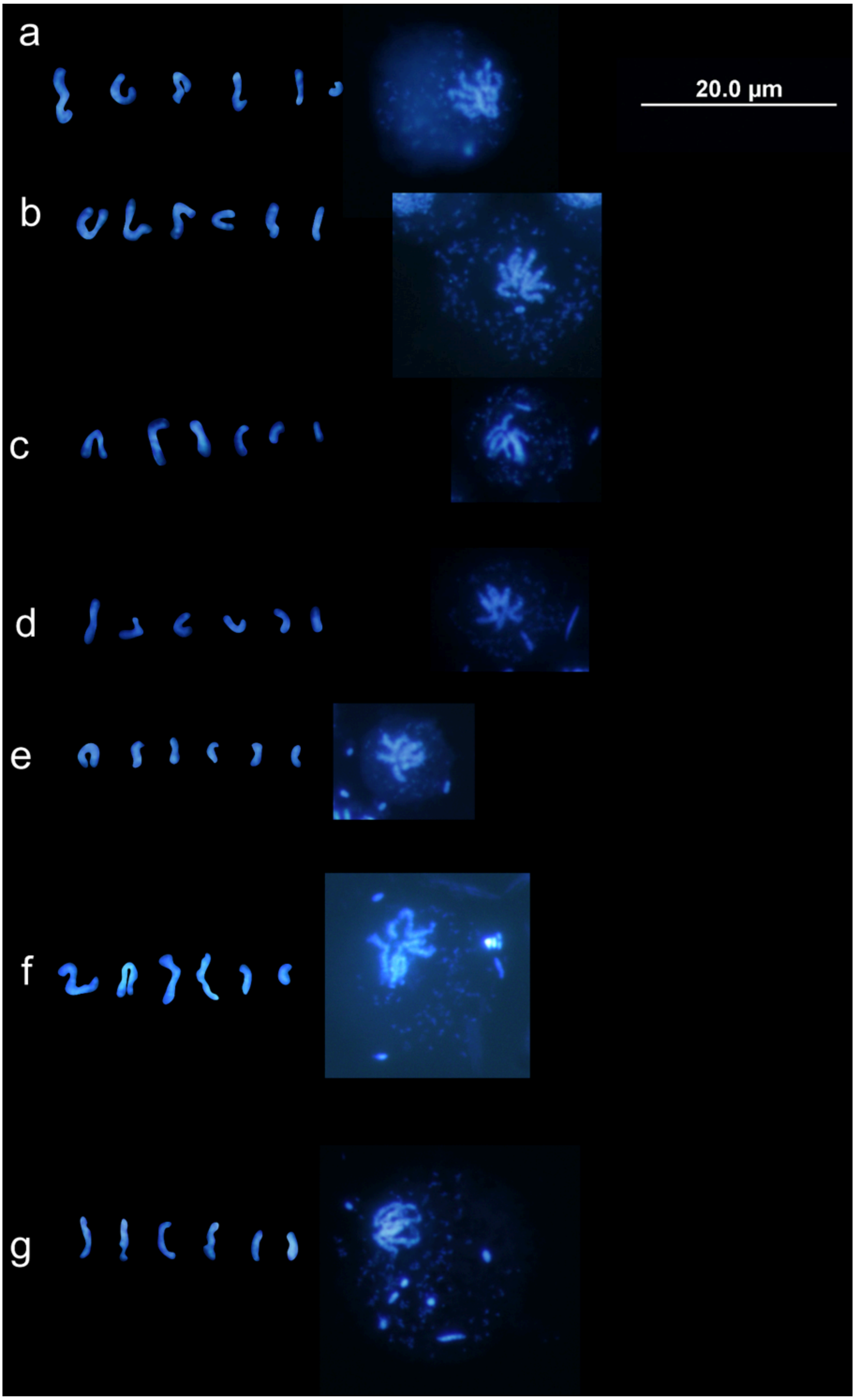

Figure S1. DAPI stained prometaphase cells (right side) with extracted expected chromosomes (left side), cells a-g correspond to cells a-g in Table S1.

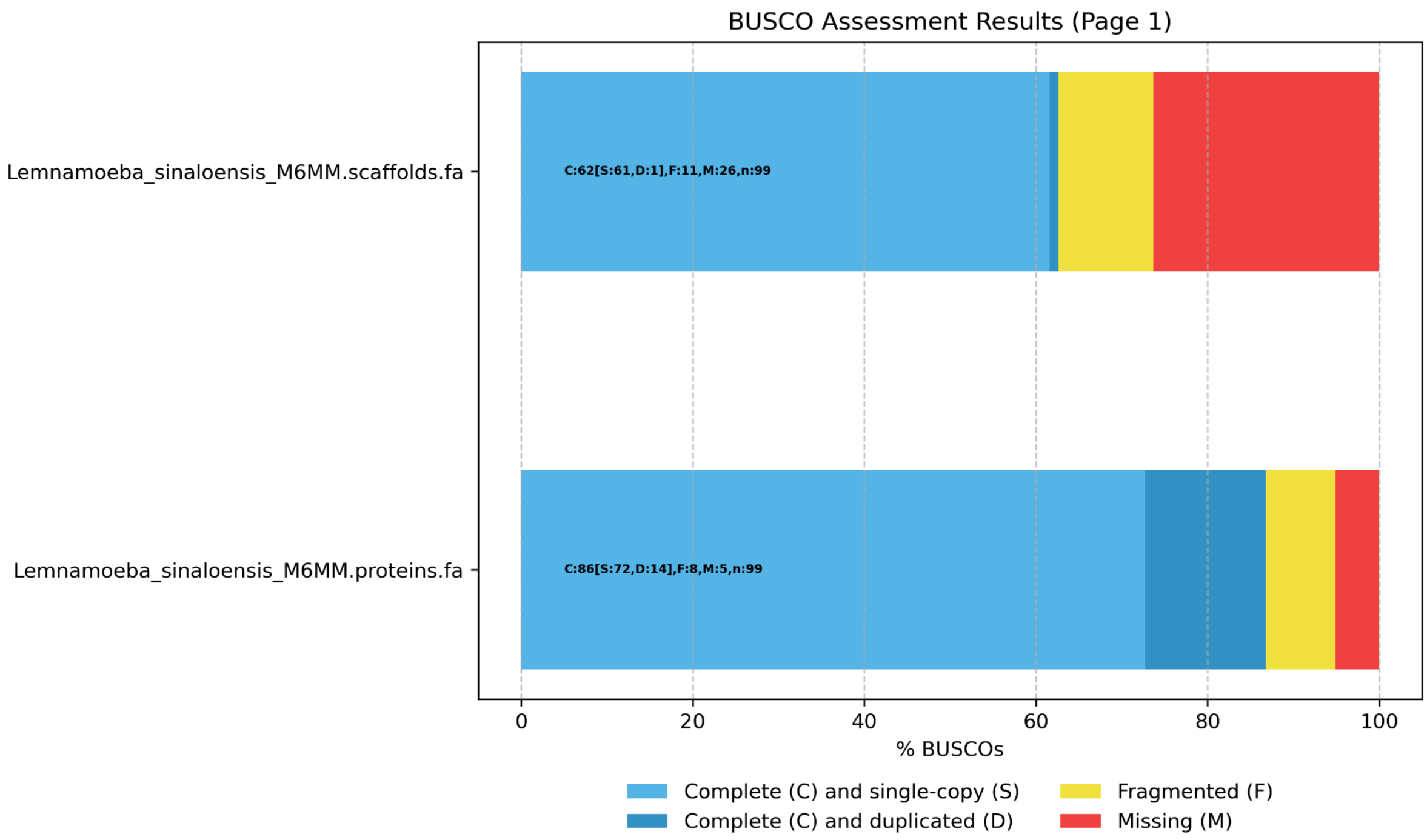

Figure S2. Completeness assessment of *L. sinaloensis* genomic sequences (top) and protein predictions (bottom) by BUSCO against the alveolata_odb12 lineage dataset.

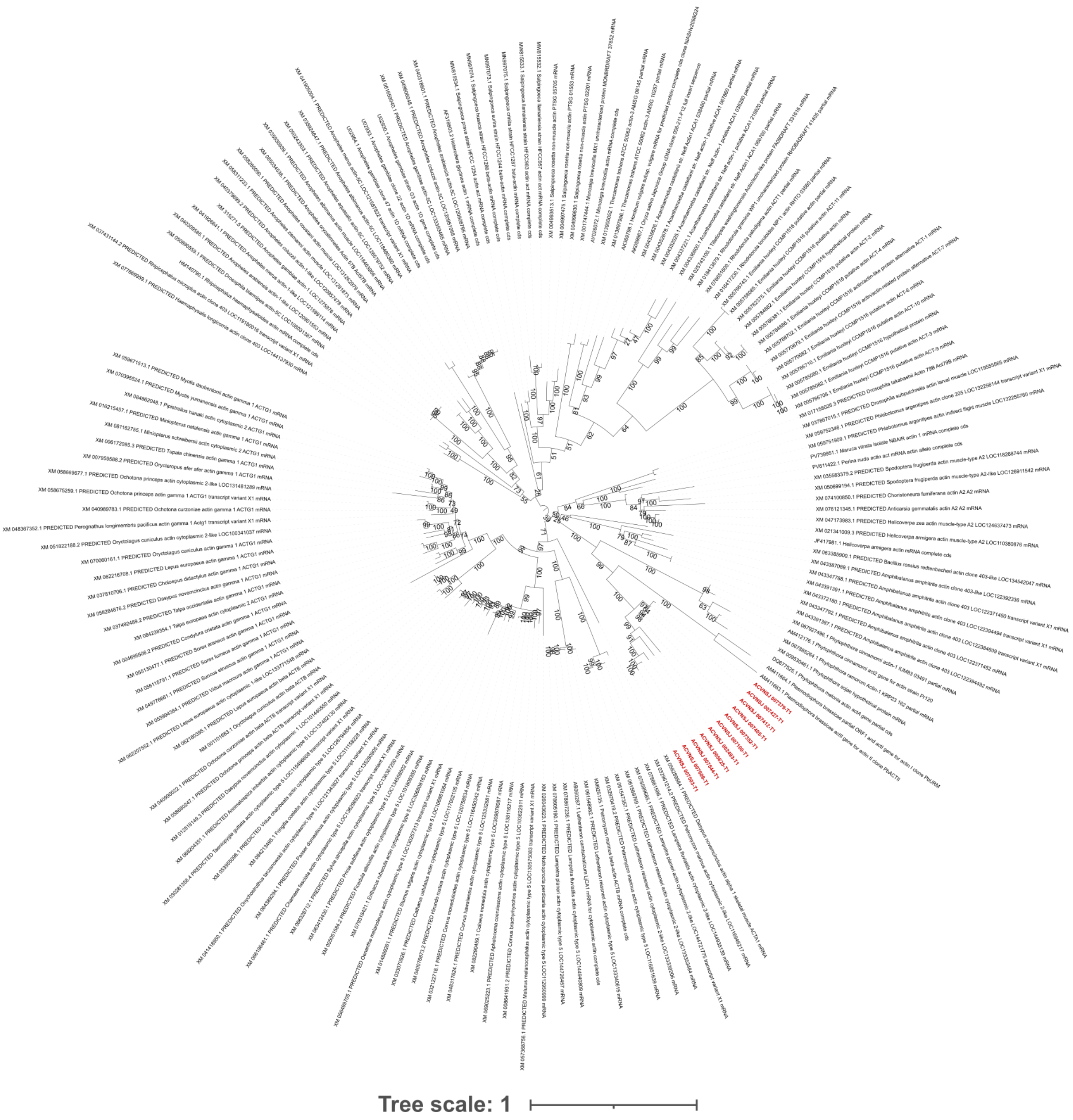

Figure S3. Maximum likelihood tree of nucleotide sequences of putative actin homologs in *Lemnamoeba sinaloensis* (red) compared to their top BLASTn hits (% identity > 83.6) in the GenBank nt database. Support values indicate ultrafast bootstrap support and the scale bar substitutions per site. The tree is mid-point rooted for visualization.

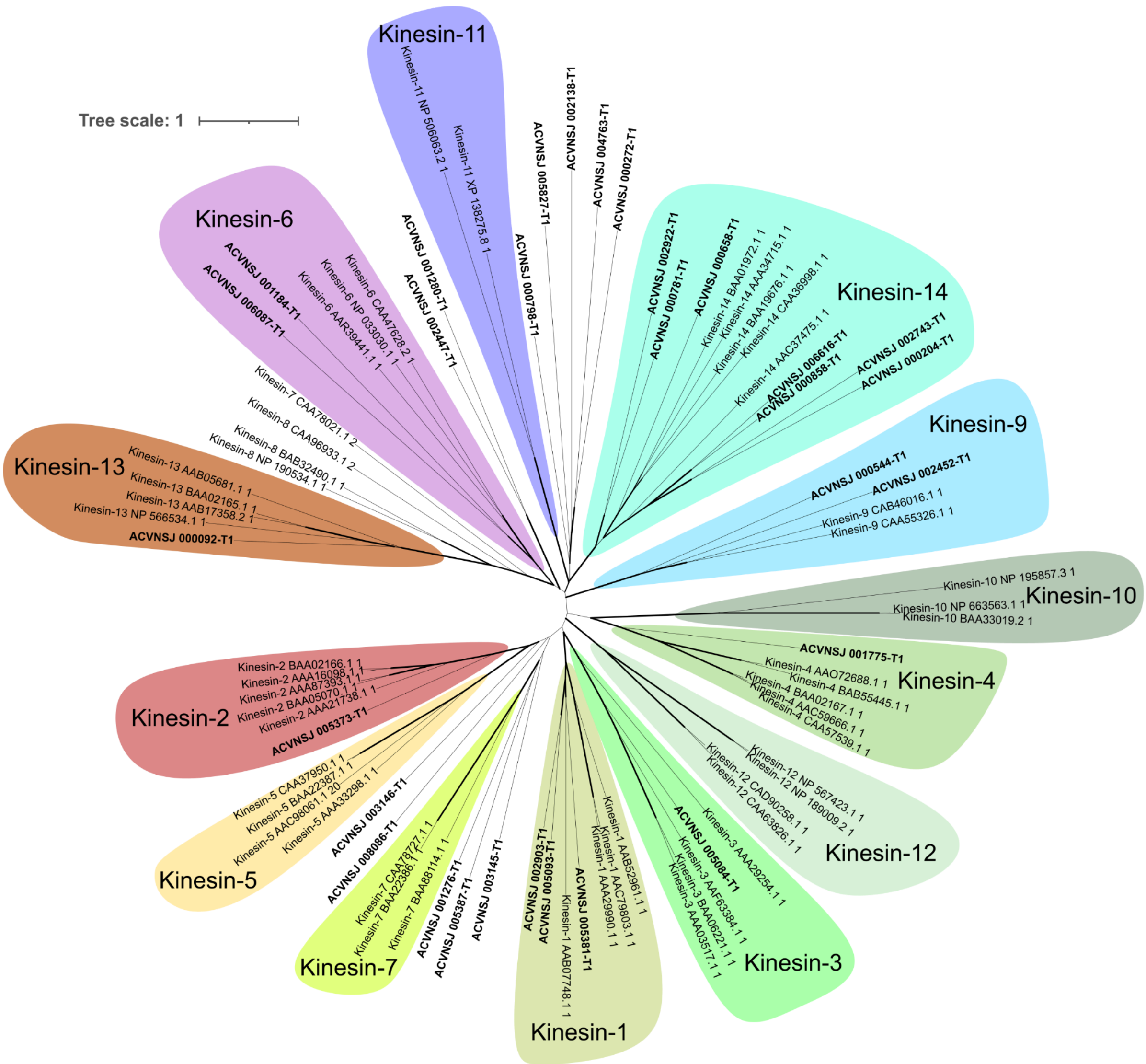

Figure S4. Maximum likelihood tree of kinesin proteins encoded in the *Lemnamoeba sinaloensis* genome compared to a reference set from Lawrence et al. (2004)[27]. Sequences in bold originate from *L. sinaloensis*. Thick branches indicate ultrafast bootstrap support >95. Note that the kinesin-8 family could not be properly resolved.

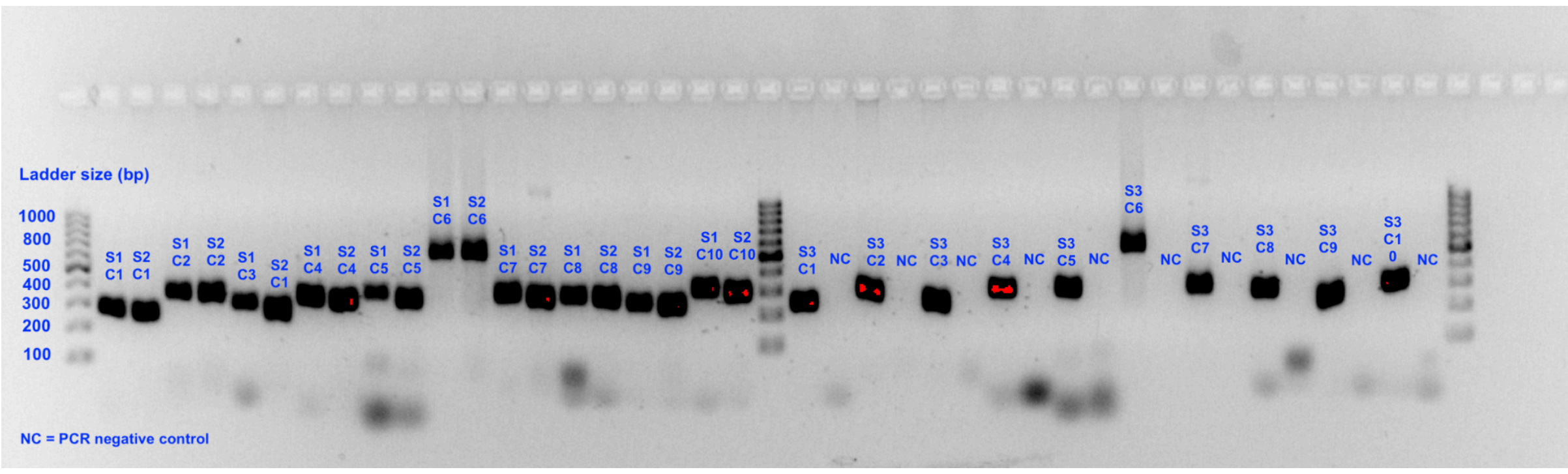

Figure S5. Electrophoresis gel showing the PCR bands that amplified the different simple-sequence repeats identified bioinformatically in the *L. sinaloensis* genome. S1, S2 and S3 correspond to three replicates of the same DNA extraction, C1-10 correspond to the primer combination according to Table S2. NC = negative control.

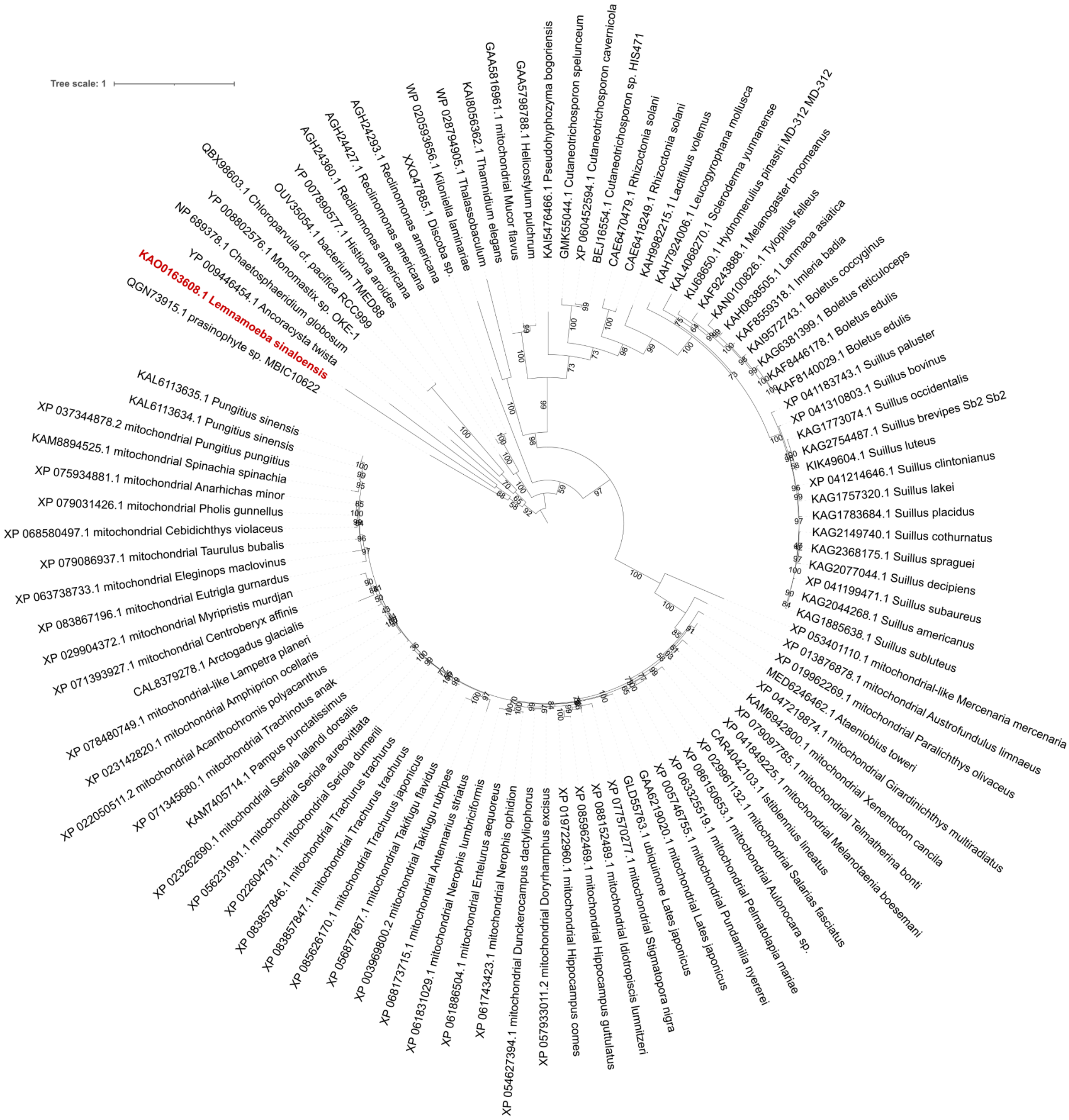

Figure S6. Maximum likelihood phylogeny of NAD9 protein models from the top 100 BLASTp hits to the nuclear-encoded *L. sinaloensis* homolog (marked in red). Support values indicate ultrafast bootstrap support and the scale bar substitutions per site. The tree is mid-point rooted for visualization. The other homologs in the clade containing the *L. sinaloensis* homolog are only mitogenome-encoded, except for one bacterial sequence.

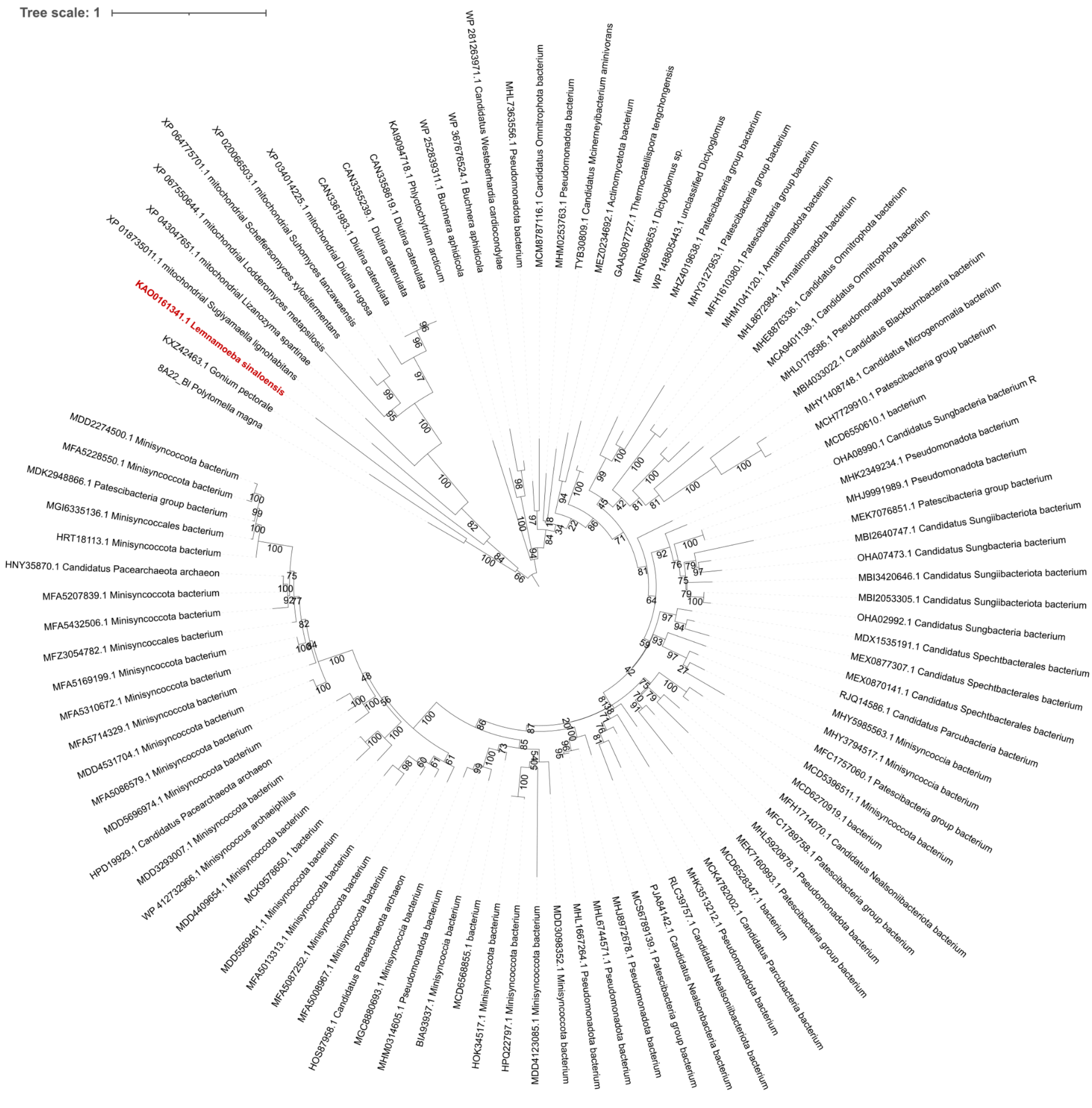

Figure S7. Maximum likelihood phylogeny of RPS12 protein models from the top 100 BLASTp hits to the nuclear-encoded *L. sinaloensis* homolog (marked in red). Support values indicate ultrafast bootstrap support and the scale bar substitutions per site. The tree is mid-point rooted for visualization. The homolog from *L. sinaloensis* clusters in a group of mitogenome-encoded genes and the remaining homologs are from bacteria (except *Phlyctochytrium arcticum*).

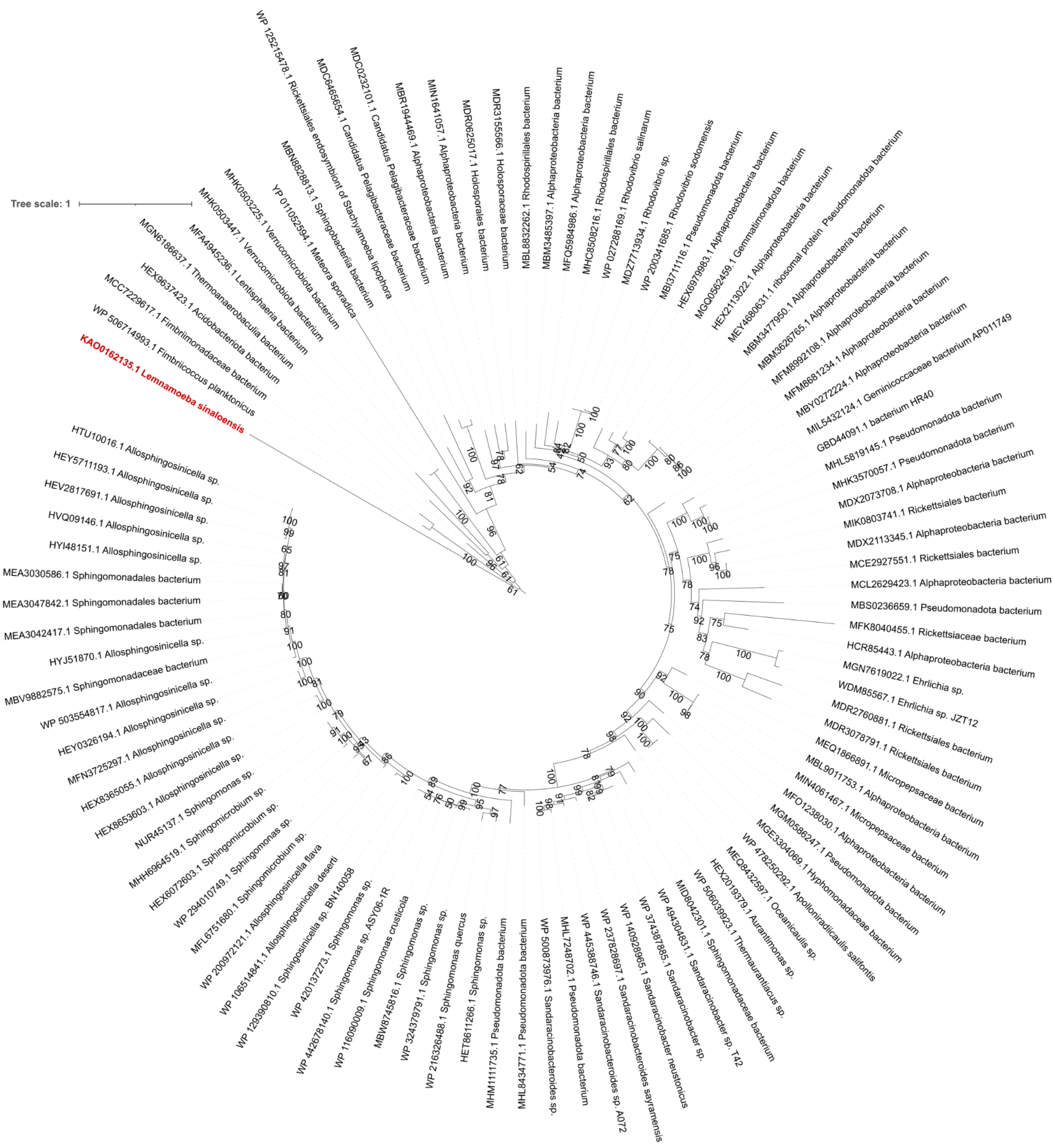

Figure S8. Maximum likelihood phylogeny of RPS4 protein models from the top 100 BLASTp hits to the nuclear-encoded *L. sinaloensis* homolog (marked in red). Support values indicate ultrafast bootstrap support and the scale bar substitutions per site. The tree is mid-point rooted for visualization. Note that all homologs are from bacteria except *L. sinaloensis* and *Meteora sporadica.*

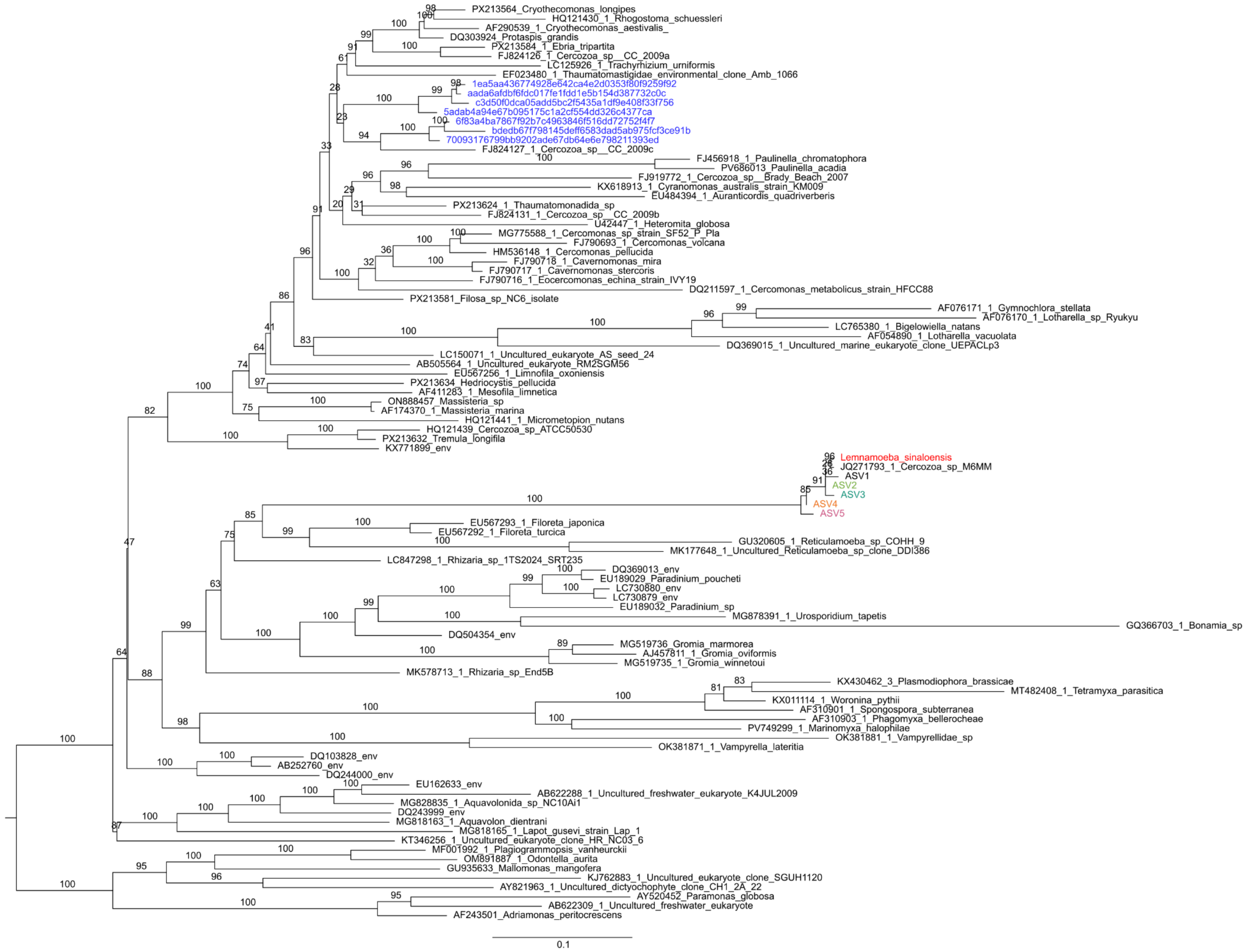

Figure S9. 18S rDNA maximum-likelihood phylogeny of *Lemnamoeba sinaloensis* and the ≥97% similar ASVs retrieved from the EukBank 18S V4 dataset. The phylogeny was inferred using IQ-TREE, and numbers on branches indicate ultrafast bootstrap support values. Blue: ASVs clustering distant from *L. sinaloensis.* ASVs clustering close to *L. sinaloensis* are renamed according to Table S7. The scale bar represents the nucleotide substitution rate.

Supplementary tables

Table S1. Measurements [µm] of the expected chromosomes from Fig. S1, cells a-g.

**Cell**	a	b	c	d	e	f	g
**Chromosome length [µm]**	6.126667	6.972	5.44527	4.308825	5.038577	6.771124	4.442238
	5.115143	6.219079	5.350857	3.64419	2.787051	6.400041	4.371083
	4.593873	4.694381	3.651016	3.576921	2.530768	5.243562	4.073637
	4.218317	4.213333	3.365556	3.17219	2.350605	5.213155	3.882097
	3.549619	3.617016	2.228159	2.583238	2.288965	2.920881	3.063192
	1.909714	3.537841	1.658698	2.293651	2.124935	2.339435	2.606061

Table S2. Identified putative homologs of *Lemnamoeba sinaloensis* cytoskeleton genes, motor protein genes, polyubiquitin, and mismatch repair genes (separate tsv file).

Table S3. Forward (F) and Reverse (R) primers and PCR settings for molecular confirmation of SSR loci. The length of the PCR product is estimated as the average of three replicates, based on measurements of the lower edge of the PCR product band in the gel image (see Fig. S5).

Name	Scaffold	Start crd	End crd	Repeat length	Primer F+R combination	Primer F name	Primer F seq	Primer R name	Primer R seq	Expected product length	Approximate length of the PCR product	Tm (Phusion; ThermoFisher calculator)
ssr1	scaffold_1	284809	284848	39	C1	ssr1.f	ACTCCTGCTCACATGGATCC	ssr1.r	CGGAGGACATGTTTTGAGGTAA	224	230	63.4
ssr2	scaffold_1	6465072	6465154	82	C2	ssr2.f	CCTCTTACTCCCCGTACCCT	ssr2.r	CGAGTGCGGAACATGTACAT	241	300	63.3
ssr3	scaffold_2	1810876	1810933	57	C3	ssr3.f	ACAGCAGCAGTAGGTACCTC	ssr3.r	CATCGGATCTCTCTGCCCTT	200	216	64.1
ssr4	scaffold_3	91649	91667	18	C4	ssr4.f	CGAGCAGTGCCTCCTCTTTT	ssr4.r	GTGCCGAGTTCCAATGGTAC	245	300	63.9
ssr5	scaffold_3	4006079	4006148	69	C5	ssr5.f	CTGTTCCACCTGCTCCTGG	ssr5.r	TGTACCAGCAATGCGAACAG	224	300	63.9
ssr6	scaffold_5	671435	671690	255	C6	ssr6.f	GCTTCCCGGACACCTACATA	ssr6.r	TGACTCACCTCTCACACATCA	377	500	63.7
ssr8	scaffold_2	397286	397379	93	C7	ssr8.f	GTCGCAGTCAGTTCTTTGGG	ssr8.r	CGCACACACAGACTACCATG	200	300	63.8
ssr9	scaffold_2	2632047	2632095	48	C8	ssr9.f	GGCGCTCCATATTGATGTTGT	ssr9.r	AACAATCTTAGTGGGCGGGA	209	300	64.1
ssr10	scaffold_3	2041989	2042057	68	C9	ssr10.f	CGATGGCAGCGTCATATCAG	ssr10.r	TAGAAACTCCTCGCACGCTT	180	200	63.8
ssr12	scaffold_2	752334	752392	58	C10	ssr12.f	CCTTGTGCGATTTAACCCGT	ssr12.r	TCGGCCGGAAGAGAAAACTA	232	300	63.9

Table S4. Results of InterProScan search with ORFs from the *Lemnamoeba sinaloensis* mitogenome as queries (separate tsv file).

Table S5. Putative genes that have been transferred from the mitogenome to the nuclear genome through endosymbiotic gene transfer, their likelihood for carrying a mitochondrial targeting peptide (MTP) and accession numbers of protein sequences from *Plasmodiophora brassicae* that were used as queries during BLAST searches. NA: not applicable.

Gene	L. sinaloensis gene model	Nuclear genome location	MTP likelihood	Pl. brassicae gene model (acc. nr.)
nad9	ACVNSJ_004873	scaffold_3	0.9348	SYZ47168
rps4	ACVNSJ_005942	scaffold_4	0.8905	SYZ47134
rps8	ACVNSJ_007793	scaffold_6	0.4987	SYZ47138
rps12	ACVNSJ_007179	scaffold_5	0.9153	SYZ47136
rps13	ACVNSJ_008289	scaffold_7	0.1187	SYZ47141
rpl14	ACVNSJ_005548	scaffold_3	0.1802	SYZ47151
rps3	No hits	No hits	NA	SYZ47147
rps7	No hits	No hits	NA	SYZ47135
rps9	NA	NA	NA	Not annotated/missing
rps10	No hits	No hits	NA	SYZ47126
rps11	No hits	No hits	NA	SYZ47140
rps14	No hits	No hits	NA	SYZ47139
rpl5	No hits	No hits	NA	SYZ47152
rpl6	No hits	No hits	NA	SYZ47166
rpl16	No hits	No hits	NA	SYZ47148
nad4L	No hits	No hits	NA	SYZ47158
nad2	No hits	No hits	NA	SYZ47125
nad6	No hits	No hits	NA	SYZ47130
atp8	No hits	No hits	NA	SYZ47150

Table S6. Amplified sequence variants showing similarity to the *Lemnamoeba sinaloensis* 18S V4 rDNA (separate tsv file).

Table S7. Original EukBank amplicon sequence variant (ASV) identifiers and their corresponding simplified names used throughout the biogeographic distribution analyses.

**Eukbank name**	**Renamed**
67979c40272eac9966eb759e6673671b12cf2d27	Lemnamoeba related asv1
f57714ffce3df4c25df0cc4045d71086a82eb5c4	Lemnamoeba related asv2
2ca3e2d13a8691c548b20a86d270d2df9807f9f4	Lemnamoeba related asv3
38ee549aeb136514faa8066a30dc9eaaa5d53b71	Lemnamoeba related asv4
37b8a3088b48540df95c352ee17dc9d87a4373c7	Lemnamoeba related asv5
